# Comparative transcriptome analysis reveals ectopic delta-5 and delta-6 desaturases enhance protective gene expression upon *Vibrio vulnificus* challenge in Tilapia (*Oreochromis niloticus*)

**DOI:** 10.1186/s12864-021-07521-5

**Published:** 2021-03-22

**Authors:** Pin-Yang Tu, Shin-Jie Huang, Venugopal Rajanbabu, Jen-Leih Wu, Jyh-Yih Chen

**Affiliations:** 1 Marine Research Station, Institute of Cellular and Organismic Biology, Academia Sinica, 23-10 Dahuen Rd., Jiaushi, Ilan, 262 Taiwan; 2 Institute of Cellular and Organismic Biology, Academia Sinica, 128 Academia Road, Section 2, Nankang, Taipei, 115 Taiwan; 3 Department of Plant Breeding 7 Genetics, Anbil Dharmalingam Agricultural College & Research Institute, Tamil Nadu Agricultural University, Tiruchirapalli, Tamil Nadu 620027 India; 4 The iEGG and Animal Biotechnology Center, National Chung Hsing University, Taichung, 402 Taiwan

**Keywords:** Delta-6 desaturase and delta-5 desaturase (D56), D56 transgenic tilapia fish, RNA-seq, Fatty acid-associated genes, Immune responsive genes, Inflammatory genes

## Abstract

**Background:**

Tilapia (*Oreochromis niloticus*) cultures are frequently infected by *Vibrio vulnificus,* causing major economic losses to production units. Previously, tilapia expressing recombinant delta-5 desaturase and delta-6 desaturase (D56) were found to be resistant to *V. vulnificus* infection. In this report, we profile the D56-mediated molecular changes underlying this resistance in tilapia. A comparative transcriptome analysis was performed on *V. vulnificus*-infected wild-type and D56-transgenic tilapia using Illumina’s sequencing-by-synthesis approach. Gene enrichment analysis on differentially expressed unigenes was performed, and the expression patterns were validated by real-time PCR.

**Results:**

Comparative transcriptome analysis was performed on RNA-sequence profiles obtained from wild-type and D56-transgenic tilapia at 0, 6 and 24 h post-infection with *V. vulnificaus*. GO and KEGG gene enrichment analyses showed that D56 regulates several pathways and genes, including fatty acid (FA) metabolism associated, and inflammatory and immune response. Expression of selected FA metabolism-associated, inflammatory and immune responsive genes was validated by qPCR. The inflammatory and immune responsive genes that are modulated by FA-associated D56 likely contribute to the enhanced resistance against *V. vulnificus* infection in Tilapia.

**Conclusions:**

Transcriptome profiling and filtering for two-fold change variation showed that 3795 genes were upregulated and 1839 genes were downregulated in D56-transgenic tilapia. These genes were grouped into pathways, such as FA metabolism, FA elongation, FA biosynthesis, biosynthesis of unsaturated FA, FA degradation, inflammation, immune response, and chemokines. FA-associated genes and immune-related genes were modulated by D56 at 6 h and 24 h post infection with *V. vulnificus*. The expression patterns of FA-related genes, inflammatory genes, antimicrobial peptide genes and immune responsive genes at 0, 3, 6, 12, 24 and 48 h post-infection suggests these genes are involved in the enhanced resistance of D56 transgenic tilapia to *V. vulnificus*.

**Supplementary Information:**

The online version contains supplementary material available at 10.1186/s12864-021-07521-5.

## Background

Tilapia (*Oreochrombis niloticus*) is an important commercial aquaculture species throughout the world, and its production is severely affected by the pathogenic bacteria *Vibrio vulnificus*, which causes septicemia in fish and humans [1–4]. Omega-3 polyunsaturated fatty acids (n-3 PUFAs) are known to exert beneficial effects, such as protection of liver, reduction of cholesterol, lower blood pressure and protect from cardiovascular diseases [5, 6]. Furthermore, n-3 PUFAs show positive ionotropic effects and minimize tachyarrhythmia in animal models [7]. Many of these effects may be mediated by alterations in the pro-inflammatory cytokines, TNF-α, IL-1β, IL-6, prostaglandin (PG) E2, and PGF1α, which modulate the immune response in model organisms [8–10]. Dietary supplementation with eicosanoids and n-3 PUFAs is well documented to affect immune cell function and B-cell activity [11, 12], and a recent report showed that PUFA-rich food limit pathogen infection in the aquatic organisms [13]. Similarly, transgenic expression of n-3 PUFA biosynthesis genes from Atlantic salmon, i.e., Fatty acyl desaturase synthase delta (*Fadsd*)*5* and *Fadsd6*, in zebrafish limits infection with *Vibrio alginolyticus* and *V. vulnificus* [5, 14].

Previously we reported the dual expression of Ss*Fadsd5* and Ss*Fadsd6* (D56) in tilapia [15]. The dual expression of these genes is under the control of a TRE-regulated CMV minimal promoter, which drives expression of D56 in liver and muscle [15]. Expression of D56 in tilapia enhances resistance to *V. vulnificus* infection [15]. In addition, the D56 transgenic tilapia exhibit altered gut microbial profiles [15]. However, the underlying molecular mechanism involved in the resistance to *V. vulnificus* has not been studied using a transcriptomic approach.

We compared the liver transcriptomes between *V. vulnificus*-susceptible wild-type tilapia and D56 transgenic tilapia with enhanced resistance to the pathogen to reveal the particular genes responsible for the resistance [15, 16]. The alterations in expression of key genes were identified by gene enrichment analysis with KEGG pathway and GO tools. We showed the involvement of fatty acid (FA)-associated genes and immunomodulatory genes in the development of resistance against *V. vulnificus* infection in tilapia.

## Results

### Expression of recombinant delta-6 desaturase and delta-5 desaturase alters the transcriptome in tilapia

**Fig. 1:**
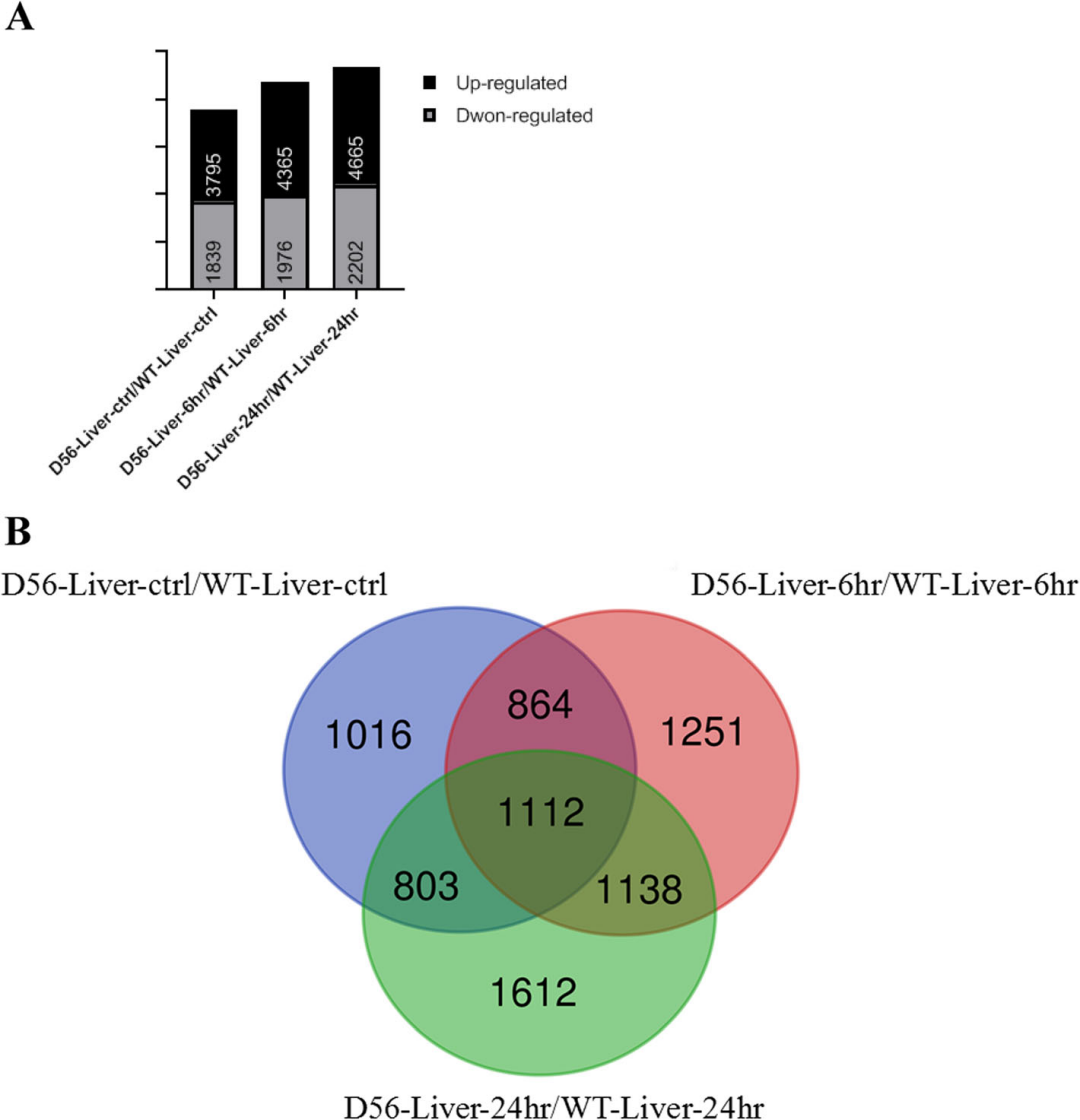
Delta 5 and Delta6 transgenic (D56) tilapia fish alters the transcriptome profile. The comparative transcriptome data between wild-type / Delta 5 and Delta6 (D56) transgenic tilapia fish were mapped and differentially expressed genes were counted. **a** Differentially expressed genes with more than or equal to two fold change have been listed. Wild-type and D56 transgenic tilapia fish liver RNA were compared in 0, 6 and 24 h post infection with *V. vulnificus*. **b** Differentially expressed genes between wild-type and D56 transgenic tilapia fish at 0, 6 and 24 h *V. vulnificus* post infected liver RNA. The number of the genes unique to specific infection condition and the number of genes commonly shared by two or three infection conditions are mentioned in the respective intersections

Wild-type and D56-transgenic tilapia were infected with *V. vulnificus*, and RNA was extracted from liver at 0, 6 and 24 h post-infection (hpi). Transcriptome sequencing of six groups of samples produced a total of 275,304,348 raw reads for wild-type and D56-transgenic tilapia. After filtering the data 48,315,226, 38,578,158 and 35,079,100 clean reads were obtained for wild-type tilapia fish at 0, 6 and 24 h infected samples, respectively, representing 92.12, 89.24 and 92.19% of raw reads (Table 1). Similarly, 37,449,898, 49,652,212 and 50,987,302 clean reads (90.62, 88.48 and 91.06% of raw reads) were obtained for D56-transgenic tilapia for 0, 6 and 24 hpi samples, respectively (Table 1). A total of 42,622 unigenes were identified from the RNA-sequencing and filtered for two-fold change in expression between *V. vulnificus* challenged wild-type and D56-transgenic tilapia (Supplementary Figure S1). At the 0 h time-point, 3795 genes were upregulated and 1839 genes were downregulated in D56-transgenic tilapia (Fig. 1a). At 6 hpi, 4365 genes were upregulated and 1976 genes were downregulated (Fig. 1a). At 24 hpi, 4665 were upregulated and 2202 genes were downregulated (Fig. 1a). We could recognize the relevance of DEG between wild-type and D56-transgenic tilapia at different time-points. We found that 1112 DEG existed at three time-points (Fig. 1b).

**Table 1:** Details of RNA sequence read

Sample	Clean	Filtered	Mapping rate
	Reads	Reads	(%)
WT-Liver-ctrl	50,952,012	48,315,226	**92.12**
WT-Liver-6 h	40,765,142	38,578,158	**89.24**
WT-Liver-24 h	36,947,506	35,079,100	**92.19**
D56-Liver-ctrl	39,667,224	37,449,898	**90.62**
D56-Liver-6 h	52,706,028	49,652,212	**88.48**
D56-Liver-24 h	54,266,436	50,987,302	**91.06**

RNA depletion of rRNA and organelle RNA was extracted from liver samples of wild-type and transgenic Tilapia fish expressing delta-6 desaturase plus delta-5 desaturase (D56) at 0, 6 and 24 h *V. vulnificaus* post infected conditions. The RNA was subjected to multiplexed RNA sequence. The total number of clean reads, filtered reads and RNA mapped reads for the six groups were shown in the table

### Fatty acid-associated genes are altered in D56-transgenic tilapia according to KEGG pathway analysis

Gene enrichment analysis using the KEGG pathway database showed a total of 24, 30 and 33 pathways were affected in D56-transgenic tilapia at 0, 6, and 24 hpi, respectively (Table 2, 3 4). Immediately after infection, altered expression of various FA-associated pathways, such as FA metabolism, FA elongation, FA biosynthesis, biosynthesis of unsaturated FA, and FA degradation were observed (Table 2). Differences in the FA degradation pathway were also observed between wild-type and D56-transgenic fish at 6 h post infection (Table 3), and the FA metabolism and FA degradation pathways were identified as differentially expressed at 24 hpi (Table 4).

**Table 2:** KEGG pathway enrichment analysis of wild-type and transgenic tilapia fish expressing delta-6 desaturase plus delta-5 desaturase (D56)

Pathway ID	Pathway	DEGs with pathway annotation	Corrected *P*-value
onl00620	Pyruvate metabolism	9	1E-07
onl01212	Fatty acid metabolism	9	7E-06
onl00010	Glycolysis / Gluconeogenesis	9	2E-05
onl03320	PPAR signaling pathway	9	3E-05
onl00071	Fatty acid degradation	7	3E-05
onl00561	Glycerolipid metabolism	8	6E-05
onl00062	Fatty acid elongation	6	0.0002
onl01040	Biosynthesis of unsaturated fatty acids	6	0.0003
onl00983	Drug metabolism - other enzymes	8	0.0005
onl00982	Drug metabolism - cytochrome P450	6	0.0014
onl00564	Glycerophospholipid metabolism	8	0.0015
onl00980	Metabolism of xenobiotics by cytochrome P450	6	0.0015
onl00480	Glutathione metabolism	7	0.0019
onl00410	beta-Alanine metabolism	4	0.0039
onl00020	Citrate cycle (TCA cycle)	4	0.0047
onl00330	Arginine and proline metabolism	5	0.0058
onl00061	Fatty acid biosynthesis	3	0.01
onl04910	Insulin signaling pathway	8	0.0133
onl00340	Histidine metabolism	3	0.0133
onl00280	Valine, leucine and isoleucine degradation	4	0.0208
onl04920	Adipocytokine signaling pathway	5	0.0257
onl00053	Ascorbate and aldarate metabolism	3	0.0326
onl00052	Galactose metabolism	3	0.0389
onl00270	Cysteine and methionine metabolism	4	0.0396

**Table 3:** KEGG pathway enrichment analysis of wild-type and transgenic tilapia fish expressing delta-6 desaturase plus delta-5 desaturase (D56) infected with *V. vulnificus* for 6 h

Pathway ID	Pathway	DEGs with pathway annotation	Corrected *P*-value
onl00983	Drug metabolism - other enzymes	10	7E-06
onl00330	Arginine and proline metabolism	8	1E-05
onl00620	Pyruvate metabolism	6	0.0001
onl00561	Glycerolipid metabolism	7	0.0003
onl00380	Tryptophan metabolism	6	0.0003
onl00100	Steroid biosynthesis	4	0.0004
onl00500	Starch and sucrose metabolism	5	0.0006
onl00982	Drug metabolism - cytochrome P450	6	0.001
onl00340	Histidine metabolism	4	0.0011
onl00480	Glutathione metabolism	7	0.0013
onl00071	Fatty acid degradation	5	0.0015
onl00010	Glycolysis / Gluconeogenesis	6	0.0029
onl00410	beta-Alanine metabolism	4	0.003
onl00053	Ascorbate and aldarate metabolism	4	0.004
onl01230	Biosynthesis of amino acids	6	0.0044
onl04145	Phagosome	11	0.006
onl00980	Metabolism of xenobiotics by cytochrome P450	5	0.0061
onl00430	Taurine and hypotaurine metabolism	3	0.0082
onl00220	Arginine biosynthesis	3	0.0091
onl00260	Glycine, serine and threonine metabolism	4	0.0091
onl04060	Cytokine-cytokine receptor interaction	11	0.011
onl03060	Protein export	3	0.0121
onl01200	Carbon metabolism	7	0.0131
onl00280	Valine, leucine and isoleucine degradation	4	0.0165
onl04672	Intestinal immune network for IgA production	6	0.0199
onl04350	TGF-beta signaling pathway	6	0.027
onl00520	Amino sugar and nucleotide sugar metabolism	4	0.028
onl00270	Cysteine and methionine metabolism	4	0.0318
onl00310	Lysine degradation	4	0.0449
onl00360	Phenylalanine metabolism	2	0.0454

**Table 4:** KEGG pathway enrichment analysis of wild-type and transgenic tilapia fish expressing delta-6 desaturase plus delta-5 desaturase (D56) infected with *V. vulnificus* for 24 h

Pathway ID	Pathway	DEGs with pathway annotation	Corrected *P*-value
onl00010	Glycolysis / Gluconeogenesis	17	2E-09
onl01200	Carbon metabolism	17	1E-05
onl04146	Peroxisome	13	3E-05
onl00071	Fatty acid degradation	9	4E-05
onl01230	Biosynthesis of amino acids	12	5E-05
onl03320	PPAR signaling pathway	11	0.0002
onl00830	Retinol metabolism	9	0.0006
onl00051	Fructose and mannose metabolism	7	0.0007
onl00982	Drug metabolism - cytochrome P450	8	0.0019
onl00980	Metabolism of xenobiotics by cytochrome P450	8	0.0021
onl00590	Arachidonic acid metabolism	9	0.003
onl00640	Propanoate metabolism	5	0.0047
onl00100	Steroid biosynthesis	4	0.005
onl00330	Arginine and proline metabolism	7	0.0052
onl00410	beta-Alanine metabolism	5	0.0066
onl00650	Butanoate metabolism	4	0.0068
onl00053	Ascorbate and aldarate metabolism	5	0.0091
onl04060	Cytokine-cytokine receptor interaction	18	0.0101
onl00140	Steroid hormone biosynthesis	7	0.0108
onl00380	Tryptophan metabolism	6	0.011
onl00250	Alanine, aspartate and glutamate metabolism	6	0.0119
onl04920	Adipocytokine signaling pathway	8	0.012
onl00052	Galactose metabolism	5	0.0121
onl00350	Tyrosine metabolism	5	0.0144
onl04350	TGF-beta signaling pathway	10	0.0155
onl00620	Pyruvate metabolism	5	0.0185
onl04110	Cell cycle	10	0.0226
onl00260	Glycine, serine and threonine metabolism	5	0.0233
onl00360	Phenylalanine metabolism	3	0.0291
onl00760	Nicotinate and nicotinamide metabolism	5	0.0308
onl01212	Fatty acid metabolism	6	0.0369
onl02010	ABC transporters	5	0.0396
onl00500	Starch and sucrose metabolism	4	0.0494

### D56-transgenic tilapia exhibit altered immune-related gene expression in GO analysis

The GO enrichment analysis showed a total of 28, 23 and 35 gene sets that were differentially expressed in D56-transgenic and wild-type tilapia at 0, 6 and 24 hpi with *V. vulnificus*. Cellular component-associated GO terms, such as major histocompatibility (MHC) class II protein complexes and extracellular protein components, were altered at all the time-points examined (Table 5, 6, 7). Biological function-associated GO terms, such as defense response to bacterium, angiogenesis, immune response, antigen presenting and presentation and inflammatory response genes were also altered in D56-transgenic tilapia. Altered molecular function-related GO terms included iron ion binding protein, cytokine and chemokine activity (Table 5, 6, 7). Taken together, the GO analysis revealed that inflammatory genes, chemokine-associated genes, cytokine-associated genes, immune-related genes and iron binding protein genes are differentially regulated in D56-transgenic and wild-type tilapia after *V. vulnificus* infection (Table 5, 6, 7). We selected target genes with significant fold change, immune-related annotation and higher FPKM value for follow up research (Supplementary File 1).

**Table 5:** Gene ontology analysis of wild-type and transgenic tilapia fish expressing delta-6 desaturase plus delta-5 desaturase (D56)

	Gene ontology term	Cluster frequency	Corrected *P*-value
	**Cellular component**		
GO:0005576	extracellular region	12/392	0.000548676
GO:0042613	MHC class II protein complex	3/392	0.010994224
GO:0005882	intermediate filament	3/392	0.011603118
GO:0005887	integral component of plasma membrane	4/392	0.015772421
	**Biological process**		
GO:0006836	neurotransmitter transport	4/392	0.001251906
GO:0043066	negative regulation of apoptotic process	2/392	0.007136566
GO:0009058	biosynthetic process	3/392	0.009280905
GO:0006814	sodium ion transport	3/392	0.010404294
GO:0001525	angiogenesis	2/392	0.010473526
GO:0019882	antigen processing and presentation	3/392	0.012231075
GO:0007411	axon guidance	2/392	0.015786162
GO:0006811	ion transport	7/392	0.021426441
GO:0006955	immune response	5/392	0.027024892
GO:0007160	cell-matrix adhesion	2/392	0.027243231
GO:0006629	lipid metabolic process	3/392	0.049135178
	**Molecular function**		
GO:0005179	hormone activity	5/392	0.00069389
GO:0009055	electron transfer activity	3/392	0.001715355
GO:0016491	oxidoreductase activity	7/392	0.001873858
GO:0020037	heme binding	6/392	0.004007088
GO:0019905	syntaxin binding	2/392	0.004392611
GO:0016747	transferase activity, transferring acyl groups other than amino-acyl groups	2/392	0.006154289
GO:0016746	transferase activity, transferring acyl groups	3/392	0.006353286
GO:0005506	iron ion binding	5/392	0.008148374
GO:0005216	ion channel activity	7/392	0.00972373
GO:0004129	cytochrome-c oxidase activity	2/392	0.010473526
GO:0042626	ATPase activity, coupled to transmembrane movement of substances	3/392	0.012878182
GO:0016705	oxidoreductase activity, acting on paired donors, with incorporation or reduction of molecular oxygen	4/392	0.017329903
GO:0016702	oxidoreductase activity, acting on single donors with incorporation of molecular oxygen, incorporation of two atoms of oxygen	2/392	0.030977671

**Table 6:** Gene ontology analysis of wild-type and transgenic tilapia fish expressing delta-6 desaturase plus delta-5 desaturase (D56) infected with *V. vulnificus* for 6 h

GO number	Gene ontology term	Cluster frequency	Corrected *P*-value
	**Cellular component**		
GO:0042613	MHC class II protein complex	4/388	0.00111901
GO:0005576	extracellular region	10/388	0.005273844
GO:0000145	exocyst	2/388	0.023258951
	**Biological process**		
GO:0006879	cellular iron ion homeostasis	5/388	7.19886E-06
GO:0019882	antigen processing and presentation	4/388	0.001296795
GO:0006955	immune response	7/388	0.00147042
GO:0005975	carbohydrate metabolic process	5/388	0.006899818
GO:0006629	lipid metabolic process	4/388	0.008996727
GO:0006096	glycolytic process	2/388	0.026732255
	**Molecular function**		
GO:0004252	serine-type endopeptidase activity	12/388	3.58836E-06
GO:0004866	endopeptidase inhibitor activity	5/388	7.56213E-05
GO:0016491	oxidoreductase activity	8/388	0.00035261
GO:0003824	catalytic activity	8/388	0.000514237
GO:0016788	hydrolase activity, acting on ester bonds	3/388	0.000760589
GO:0004181	metallocarboxypeptidase activity	3/388	0.001314446
GO:0004222	metalloendopeptidase activity	5/388	0.005118182
GO:0016746	transferase activity, transferring acyl groups	3/388	0.006176615
GO:0005125	cytokine activity	2/388	0.009116399
GO:0008289	lipid binding	3/388	0.032301052
GO:0005506	iron ion binding	4/388	0.034540735
GO:0005509	calcium ion binding	9/388	0.037632531
GO:0016616	oxidoreductase activity, acting on the CH-OH group of donors, NAD or NADP as acceptor	2/388	0.040369571
GO:0004869	cysteine-type endopeptidase inhibitor activity	2/388	0.042492616

**Table 7:** Gene ontology analysis of wild-type and transgenic tilapia fish expressing delta-6 desaturase plus delta-5 desaturase (D56) infected with *V. vulnificus* for 24 h

	Gene ontology term	Cluster frequency	Corrected *P*-value
	**Cellular component**		
GO:0005576	extracellular region	20/614	3.58031E-06
GO:0005737	cytoplasm	12/614	4.79812E-05
GO:0042613	MHC class II protein complex	4/614	0.005816676
GO:0005694	chromosome	2/614	0.016811817
GO:0005667	transcription factor complex	2/614	0.027216225
GO:0005923	bicellular tight junction	4/614	0.038570306
	**Biological process**		
GO:0006096	glycolytic process	7/614	1.13304E-07
GO:0006955	immune response	14/614	4.69497E-07
GO:0006879	cellular iron ion homeostasis	6/614	3.8588E-06
GO:0019882	antigen processing and presentation	4/614	0.006685535
GO:0007160	cell-matrix adhesion	3/614	0.007455887
GO:0008610	lipid biosynthetic process	2/614	0.01454755
GO:0042742	defense response to bacterium	2/614	0.01454755
GO:0043066	negative regulation of apoptotic process	2/614	0.016811817
GO:0009116	nucleoside metabolic process	2/614	0.024421755
GO:0006954	inflammatory response	2/614	0.033166717
	**Molecular function**		
GO:0005506	iron ion binding	27/614	3.83798E-23
GO:0020037	heme binding	26/614	1.05274E-19
GO:0016705	oxidoreductase activity, acting on paired donors, with incorporation or reduction of molecular oxygen	22/614	2.62412E-19
GO:0016491	oxidoreductase activity	20/614	5.06286E-12
GO:0003824	catalytic activity	11/614	0.000162737
GO:0004674	protein serine/threonine kinase activity	8/614	0.000452088
GO:0004866	endopeptidase inhibitor activity	5/614	0.000626808
GO:0008009	chemokine activity	6/614	0.000757102
GO:0016702	oxidoreductase activity, acting on single donors with incorporation of molecular oxygen, incorporation of two atoms of oxygen	4/614	0.000867978
GO:0005125	cytokine activity	3/614	0.001448524
GO:0042626	ATPase activity, coupled to transmembrane movement of substances	4/614	0.00715045
GO:0008374	O-acyltransferase activity	2/614	0.012426999
GO:0016887	ATPase activity	5/614	0.013302119
GO:0016746	transferase activity, transferring acyl groups	3/614	0.021148212
GO:0016773	phosphotransferase activity, alcohol group as acceptor	2/614	0.021753335
GO:0016758	transferase activity, transferring hexosyl groups	3/614	0.025460073
GO:0003779	actin binding	5/614	0.029257274
GO:0004222	metalloendopeptidase activity	5/614	0.03112367
GO:0019904	protein domain specific binding	2/614	0.036314673

### Ectopic D56 alters FA metabolism-related genes

**Fig. 2:**
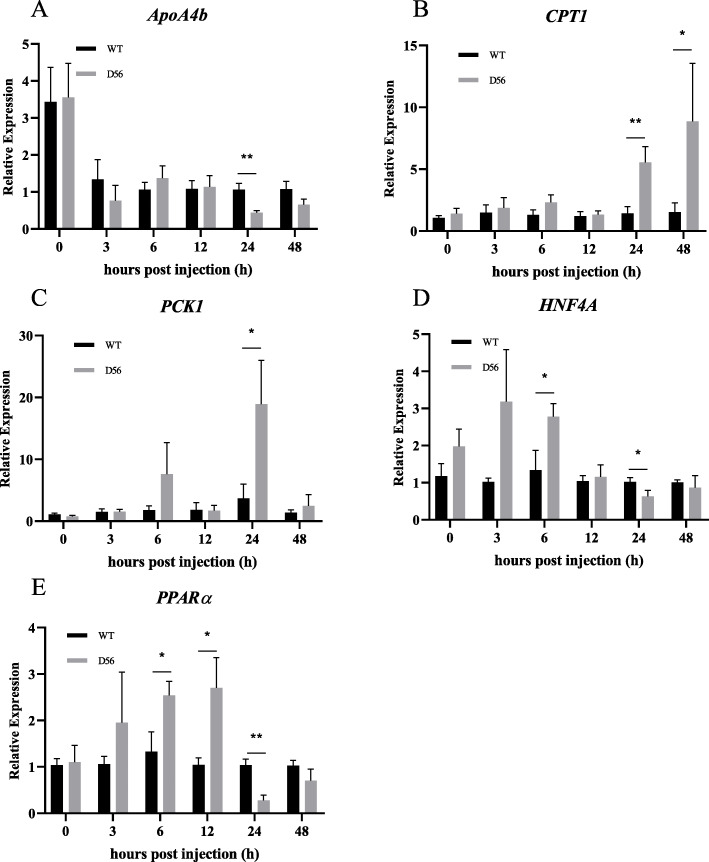
Delta 5 and Delta 6 transgenic (D56) tilapia fish modulates the expression of Fatty acid associated gene expression. Wild-type and D56 transgenic tilapia fish challenged with *V. vulnificus* and liver samples from 0, 3, 6, 12, 24 and 48 h post infected conditions were collected for qPCR. The fatty acid associated gene expression relative to *EF-1α* was estimated. **a** *ApoA4b*, **b** *CPT1*, **c** *PCK1*, **d** *HNF4A*, **e** *PPARα*. Values represented as Mean ± SEM (*n* = 5). Significance was determined by *T*-TEST (*P < 0.05,**P < 0.01,***P < 0.001)

Since the KEGG analysis showed FA-associated pathways are altered in D56-transgenic tilapia, the expression of selected FA pathway-associated genes was analyzed by real-time PCR at 0, 3, 6, 12, 24 and 48 hpi with *V. vulnificus*. Since the D56 transgenes (*delta-6 desaturase* and *delta-5 desaturase*) are associated with FA biosynthesis, we analyzed the expression pattern by qPCR at many time-points. Significant alterations in the expression of FA-associated genes were observed from the qPCR data (Fig. 2). Notably, *ApoA4b* was downregulated by D56-transgenic tilapia at 24 hpi (Fig. 2a). *CPT1* was upregulated at 24 and 48 hpi (Fig. 2b), and *PCK1* was upregulated at 24 hpi (Fig. 2c). *HNF4A* was upregulated at 6 hpi and downregulated at 24 hpi (Fig. 2d). *PPARα* was upregulated at 6 and 12 hpi, but it was downregulated at 24 hpi (Fig. 2e). These results showed that FA metabolism-related genes are altered in transgenic tilapia upon *V. vulnificus* infection.

### Ectopic D56 modulates immune response genes

In addition to FA-associated genes, several inflammatory and immune responsive genes were altered in D56-transgenic tilapia according to the GO enrichment analysis (Table 5, 6, 7). In addition, tilapia are known to express several antimicrobial peptides (AMPs), such as Tilapia Hepcidin, LEAP2, TP3, TP4, TP5 and Progranulin (PGRN), which have been reported to exert immunomodulatory functions. Hence, expression of genes associated with pro-inflammatory cytokines, immune responsive genes and AMPs were assessed at 0, 3, 6, 12, 24 and 48 hpi with *V. vulnificus* in wild-type and D56-transgenic tilapia liver.

**Fig. 3:**
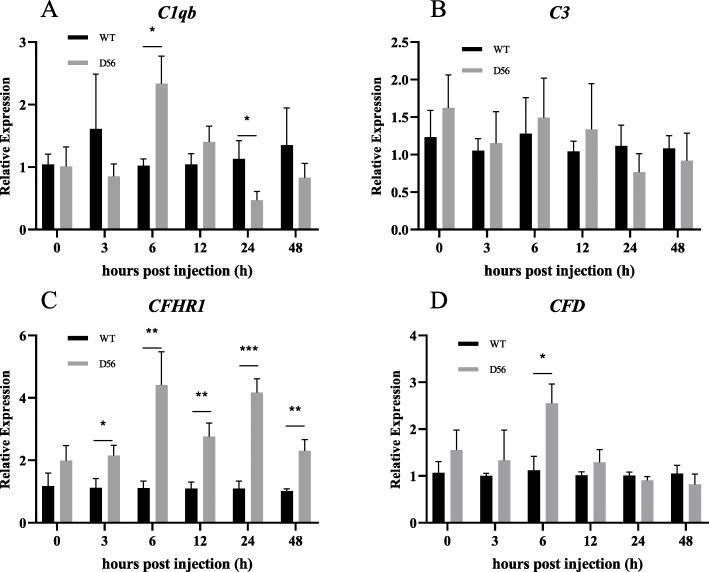
Delta 5 and Delta 6 transgenic (D56) tilapia fish alters the Complements gene expression. Wild-type and D56 transgenic tilapia fish challenged with *V. vulnificus* and liver samples from 0, 3, 6, 12, 24 and 48 h post infected conditions were collected for qPCR. The complements gene expression relative to *EF-1α* was estimated. **a** *C1qb*, **b** *C3*, **c** *CFHR1*, **d** *CFD*. Error bar is Mean ± SEM (n = 5). Significance was determined by *T*-TEST (*P < 0.05,**P < 0.01,***P < 0.001)

**Fig. 4:**
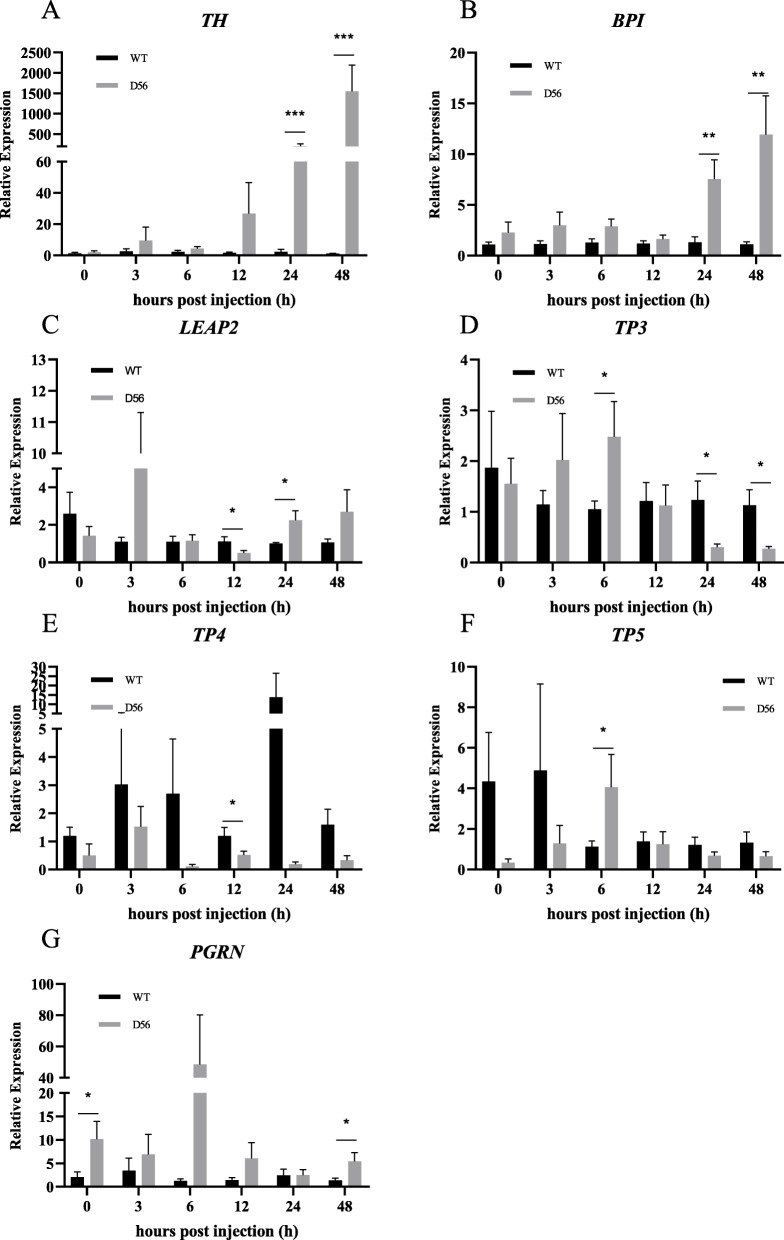
Delta 5 and Delta 6 transgenic (D56) tilapia fish alters the Antimicrobial peptide (AMP) specific gene expression. Wild-type and D56 transgenic tilapia fish challenged with *V. vulnificus* and liver samples from 0, 3, 6, 12, 24 and 48 h post infected conditions were collected for qPCR. The AMP specific gene expression relative to *EF-1α* was estimated. **a** *TH*, **b** *BP1*, **c** *LEAP2*, **d** *TP3*, **e** *TP4*, **f** *TP5*, **g** *PGRN*. Values represented as Mean ± SEM (n = 5). Significance was determined by *T*-TEST (*P < 0.05,**P < 0.01,***P < 0.001)

**Fig. 5:**
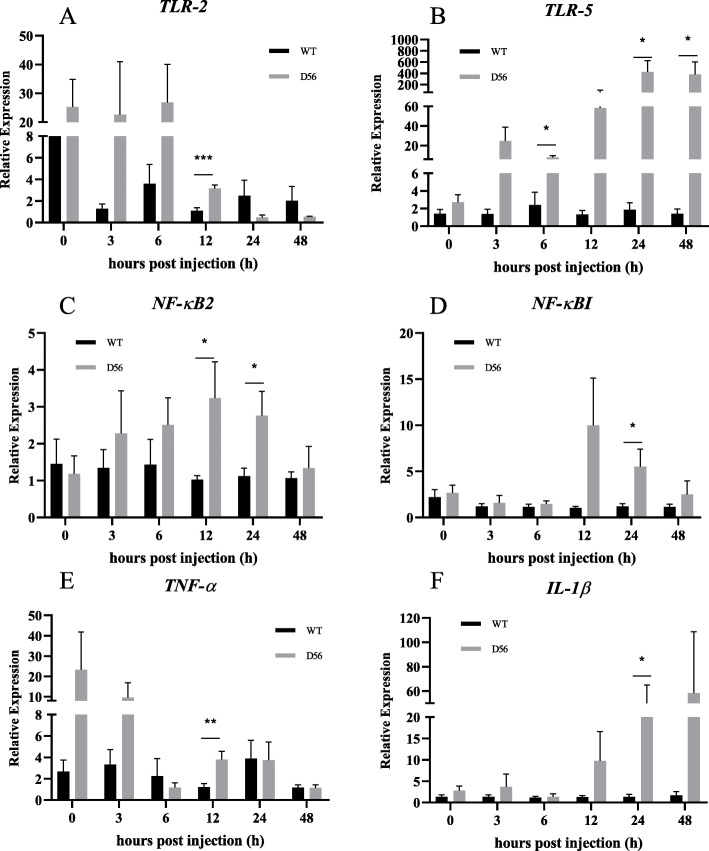
Delta 5 and Delta 6 transgenic (D56) tilapia fish modulates the pro-inflammatory cytokine gene expression. Wild-type and D56 transgenic tilapia fish challenged with *V. vulnificus* and liver samples from 0, 3, 6, 12, 24 and 48 h post infected conditions were collected for qPCR. Representative pro-inflammatory cytokine gene expression relative to *EF-1α* was estimated. **a** *TLR-2*, **b** *TLR-5*, **c** *NF-κB2*, **d** *NF-κBI*, **e** *TNF-α*, **f** *IL-1β*. Values represented as Mean ± SEM (n = 5). Significance was determined by *T*-TEST (*P < 0.05,**P < 0.01,***P < 0.001)

**Fig. 6:**
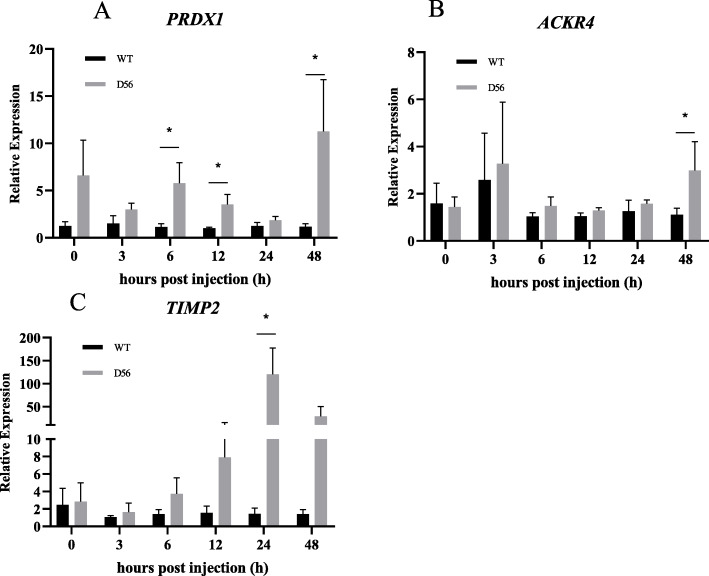
Delta 5 and Delta 6 transgenic (D56) tilapia fish alters *PRDX1*, *ACRK4* and *TIMP2* gene expression. Wild-type and D56 transgenic tilapia fish challenged with *V. vulnificus* and liver samples from 0, 3, 6, 12, 24 and 48 h post infected conditions were collected for qPCR. Expression of **a** *PRDX1*, **b** *ACKR4*, **c** *TIMP2* genes relative to *EF-1α* was estimated. Values represented as Mean ± SEM (n = 5). Significance was determined by *T*-TEST (*P < 0.05,**P < 0.01,***P < 0.001)

In D56-transgenic tilapia, the Complement C1q sub-component subunit B (*C1qb*) was upregulated at 6 hpi and downregulated at 24 hpi. Complement factor H-related protein 1 (*CFHR1*) was upregulated at 3, 6, 12, 24 and 48 hpi, and Complement factor D (*CFD*) was upregulated at 6 hpi (Fig. 3). The AMPs also showed significant differences in expression between wild-type and D56-transgenic tilapia. Tilapia Hepcidin (*TH*) was altered at 24 and 48 hpi; Binding protein I (*BPI*) was regulated at 24 and 48 hpi; liver-enriched antimicrobial peptide-2 (*LEAP2*) was altered at 12 and 24 hpi; Tilapia Piscidin (*TP*)3 was differentially expressed at 6, 24, and 48 hpi; *TP4* was altered at 12 hpi; *TP5* was altered at 6 hpi; *PGRN* expression differed at 0 and 48 hpi (Fig. 4). For the inflammatory factors, significant differences between wild-type and D56-transgenic tilapia were detected for *NF-κB2* at 12 and 24 hpi; *NF-κBI* was altered at 24 hpi; Toll-like receptor (*TLR*)*-2* was altered at 12 hpi; *TLR-5* was altered at 6, 24 and 48 hpi; Tumor necrosis factor (*TNF*)*-α* was altered at 12 hpi; Interleukin (*IL*)*-1β* was altered at 24 hpi (Fig. 5). Other immune-related genes were also affected. Peroxiredoxin (*PRDX*)*1* was altered at 6, 12 and 48 hpi; Atypical chemokine receptor (*ACKR*)*4* was altered at 48 hpi; Tissue inhibitor of metalloproteinase (*TIMP*)*2* was altered at 24 hpi (Fig. 6).

### Ectopic D56 alters pro-inflammatory cytokines and *CFD* in whole blood sample

**Fig. 7:**
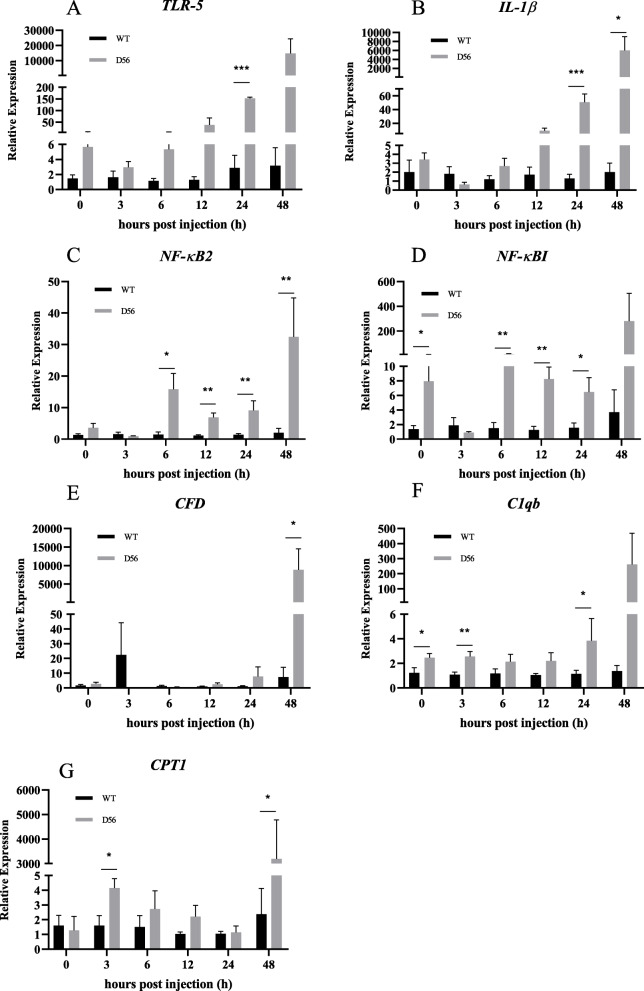
Delta 5 and Delta 6 transgenic (D56) tilapia fish modulates the pro-inflammatory cytokines and immune-related genes in whole blood. Wild-type and D56 transgenic tilapia fish challenged with *V. vulnificus* and whole blood samples from 0, 3, 6, 12, 24 and 48 h post infected conditions were collected for qPCR. Expression of **a** *TLR-2*, **b** *IL-1β*, **c** *NF-κB2*, **d** *NF-κBI*, **e** *CFD*, **f** *C1qb*, **g** *CPT1* genes relative to *EF-1α* was estimated. Values represented as Mean ± SEM (n = 5). Significance was determined by *T*-TEST (*P < 0.05,**P < 0.01,***P < 0.001)

We also measured gene expression in whole blood samples after challenge. Expression of cytokines, inflammatory factors and complement-related genes was analyzed by real-time qPCR. For inflammatory factors, cytokines and complement-related genes, we found that were several significant differences between wild-type and D56-transgenic tilapia whole blood. *TLR-5* was altered at 24 hpi; *NF-κB2* was altered at 6, 12, 24 and 48 hpi; *NF-κBI* was altered at 0, 6, 12, and 24 hpi; *IL-1β* was altered at 24 and 48 hpi; *C1qb* was altered at 0, 3 and 24 hpi; *CFD* was altered at 48 hpi (Fig. 7a-f). In whole blood samples, the expression level of *CPT1* was also different at 3 and 48 hpi (Fig. 7g). According to these results, protective and immune-related genes are induced in transgenic tilapia upon *V. vulnificus* infection.

## Discussion

Tilapia (*Oreochromis niloticus*) is a staple product of the aquaculture industry, with annual consumption exceeding 3.7 million metric tons, as of 2014 [17]. Presently tilapia are grown in fresh-water pond culture systems in approximately 125 countries [18]. Breeding programs were used to develop improved versions of tilapia with high biomass [18]. Availability of whole genome sequence and RNA sequence data in recent years has allowed a greater understanding of the genetic makeup and expression profiles of different strains or groups of tilapia [19]. In fresh-water and brackish-water cultures, tilapia is prone to infection with the aquatic bacterial pathogen, *V. vulnificus*, which severely threatens the tilapia production [4, 18, 20, 21]. *V. vulnificus* is a halophytic Gram-negative bacillus-type bacterium that can cause skin lesions, soft tissue dysfunction, and sepsis-induced mortality in tilapia or people who consume raw fish containing this pathogen [22, 23]. The *V. vulnificus* strain 93 U204 has been isolated from an infected tilapia fish and its genome was previously sequenced [3].

Regulation of gene expression plays a major role in an organisms defense against pathogens [16]. Sequencing-by-synthesis on an Illumina RNA-sequence platform has become a widely applied method for comparative transcriptome analysis [24, 25]. The primary sequence-by-synthesis data is contained in a multiplexed and mixed form with sequence information of all the groups in a Bcl file [26]. The complex Bcl form has to be converted into a readable form, such as FASTQ format prior to further analysis [14]. The comprehensive computation-based resource, Gene ontology (GO), is extensively used to analyze the large amounts of FASTQ converted reads obtained from transcriptome analysis [27, 28]. Hence, we analyzed our transcriptome data with the KEGG pathway tool to search for major pathways altered in D56 transgenic tilapia compared to wild type. The major enriched genes in identified KEGG pathways and major genes or gene groups from GO analysis were measured by real-time PCR to confirm the regulation of gene expression by D56 transgenic tilapia fish compared to wild types.

Several reports have shown that resistance to infection can be enhanced in tilapia. AMPs, such as TP3 and TP4 have been reported to decrease the bacterial counts of *V. vulnificus* [29, 30]. In addition, the multifunctional growth factor, PGRN, has been reported to modulate the immune response and improve survival of zebrafish infected with *V. vulnificus* [20]. Similarly, the granulin peptide, GRN-41, has been reported to exert anti-bacterial function against *V. vulnificus* [31]. When tilapia are fed with Epinecidin (Epi)-1-expressing transgenic Artemia, the mortality rate caused by *V. vulnificus* infection is decreased [32]. These studies demonstrate the utility of AMPs in controlling *V. vulnificus* infection.

D56-transgenic zebrafish and tilapia exhibit resistance to *V. vulnificus* infection [5, 15]. The genes expressed in D56-transgenic fish, Atlantic salmon *Fadsd5* and *Fadsd6*, play an important role in n-3 PUFA biosynthesis. Interestingly, exogenous FAs assimilated into the vibrio species affect the swimming motility, bacterial membrane structure, permeability, and virulence [33].

A comparative analysis of liver transcriptomes of wild-type and D56-transgenic tilapia infected with *V. vulnificus* was performed in this study. The multiplexed data of the six groups in the Bcl file were quality controlled, and sequence data for each group was multiplexed into FASTQ format. From the short reads, 88.48–92.19% were mapped to the RNA genome in all samples. Thus, nearly 90% of the transcriptome was mapped for the comparative analysis. The KEGG pathway analysis showed several FA-associated pathways in wild-type and D56-transgenic tilapia groups at 0 hpi with *V. vulnificus* (Table 2). KEGG analysis also revealed that the drug metabolism pathway, amino acid pathways and Cytochrome 450 pathway were altered by D56 (Table 2). These results showed that the expression of D56 enhances FA biosynthesis, especially biosynthesis of unsaturated fatty acids, and it may also lead to the modulation of additional pathways. Several such pathways were also differentially activated between wild-type and D56-transgenic fish group 6 h and 24 h post infected with *V. vulnificus* (Table 3, 4). These data demonstrated the D56-mediated FA pathway may control several amino acid biosynthesis mechanisms in addition to stress response mechanisms related to pathogen resistance (Table 2, 3, 4).

The GO analysis showed that genes associated with inflammation, chemokine synthesis, iron homeostasis and immune response are altered by D56 expression in tilapia (Table 5). The iron binding genes are related to the AMP hepcidin [34–36]. Hence, it is possible that tilapia hepcidins (TH), such as TH1–5 and TH2–3, may be functionally affected by D56 expression. Previously, the anti-*V. vulnificus* activity of TH2–3 has been reported [37]. Thus, further investigations may explore the possibility that D56 regulates tilapia hepcidins. In addition, several AMPs that are secreted by tilapia fish are associated with innate immunity and immune modulatory functions. Hence, the FA-associated D56-transgene expression may also directly alter the level of AMPs in tilapia fish. To address this possibility, the expression AMP-associated genes in tilapia was studied by qPCR (Fig. 4). *TH* and *BP1* were upregulated at 24 h and 48 hpi with *V. vulnificus* in D56-transgenic tilapia, suggesting the activation of these peptides was promoted by D56 to combat *V. vulnificus* infection (Fig. 4). The complement genes, *C1qb*, *CFHR1* and *CFD* were also upregulated in D56-transgenic tilapia after *V. vulnificus* infection (Fig. 3). It is also possible that the plasma anti-bacterial function is stimulated by D56 expression in transgenic tilapia [38, 39]. The inflammation-associated gene expression included early upregulation of *TLR-2* and *TNF-α* in D56-transgenic fish (Fig. 5). The expression level of *NF-κB* did not vary, however, it is possible that the translocation of NF-κB from cytoplasm to nucleus triggered the activation of downstream genes, such as *TNF-α* [40]. The CD8-related Peroxiredoxin (PRDX)1, G-protein receptor associated Atypical Chemokine Receptor (ACKR)4, Tissue inhibitor of Metalloprotease (TIMP)2 associated with extracellular matrix were also altered by the presence of D56 (Fig. 6). Probing the immunomodulatory gene expression in whole blood revealed that genes such as *NF-κB2*, *TLR-5*, *IL-1β*, *CFD* and *C1qb* were upregulated by D56 after *V. vulnificus* infection (Fig. 7). Altogether, the FA-associated pathways triggered in the D56-transgenic tilapia appear to regulate a variety of immune-related genes that may serve to enhance resistance to *V. vulnificus* infection.

## Conclusions

**Fig. 8:**
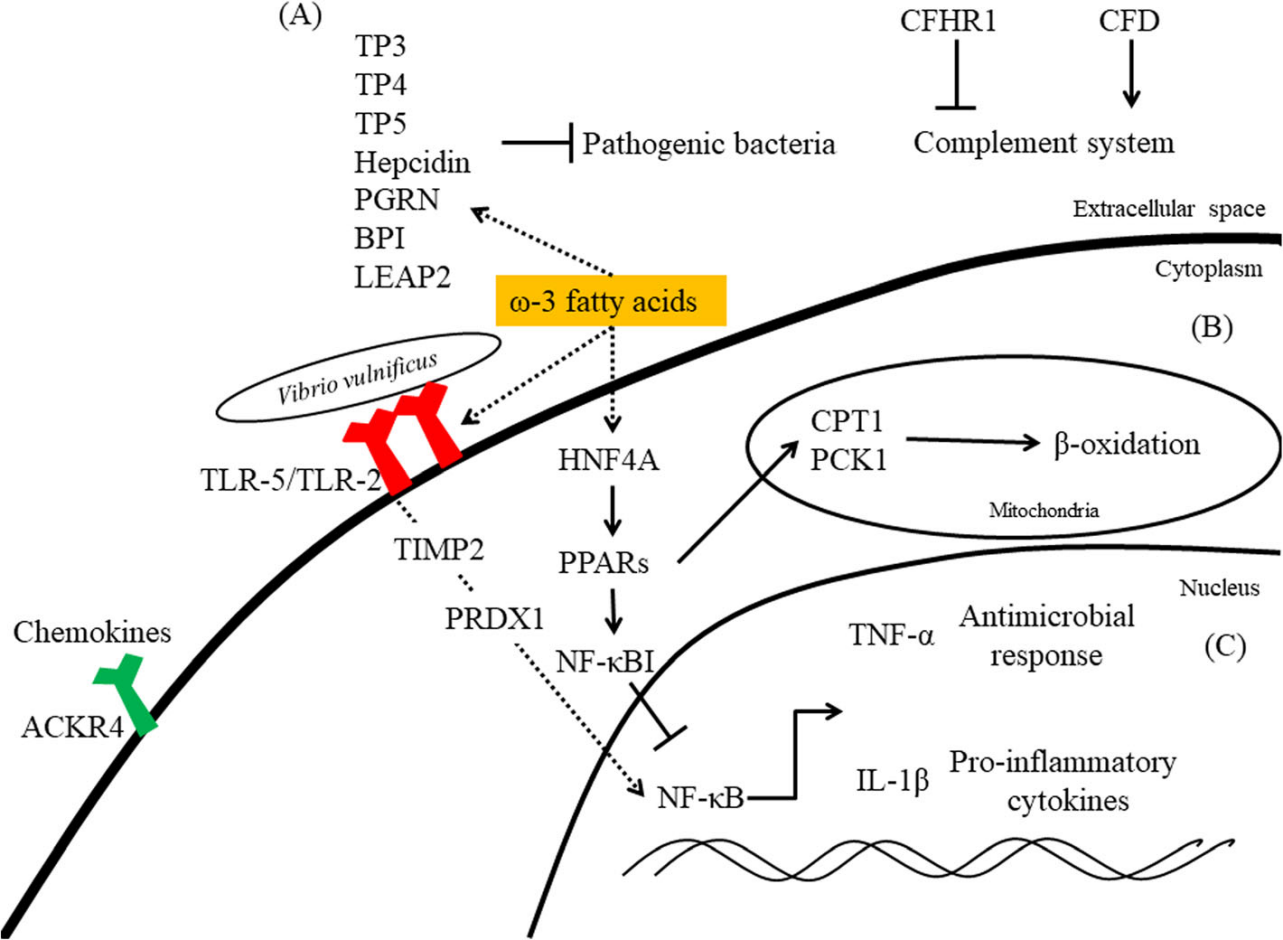
Proposed mechanism of Delta 5 and Delta 6 transgenic (D56) mediated ω-3 fatty acid metabolism to enhance resistance against *V. vulnificus* in Tilapia. **a** The ω-3 fatty acids synthesized in D56 transgenic fish regulates or activates the extra cellular acting agents such as Antimicrobial peptide (AMP), Complement system and ACKR4 by altering their expression. **b** In cytosol D56 mediated ω-3 fatty acid regulates HNF4A, TLR-5 and TLR-2, PPARs, TIMP2 and PRDX1. **c** In nucleus transcription factors such as NF-κB is altered to regulate the proinflammatory cytokines. (Image from ourselves)

We compare the results between NGS and qPCR (Supplementary Table S2). We found that *TLR-2*, *TLR-5* and *ACKR4* were induced in the D56-transgenic line. TLR-5 and TLR-2 may stimulate a pro-inflammatory response, and ACKR4 blocks chemokine signaling. Through the regulation of *CFHR1* and *CFD*, n-3 PUFAs can modulate the complement system. Moreover, AMP-associated genes, including *TP3*, *TP4*, *TP5*, *TH*, *PGRN*, *BPI* and *LEAP2*, may possibly be regulated by n-3 PUFAs (Fig. 8a). In terms of downstream effects, *HNF4A* and *PPARs* may induce *CPT1* and *PCK1*. This action would enhance ATP production from FA oxidation and help the host overcome infection by pathogenic bacteria (Fig. 8b). Furthermore, *NF-κB* downstream genes, such as *TNF-α* and *IL-1β*, may suppress pathogens (Fig. 8c). In this study, we measured the transcriptome-wide responses of wild-type and D56-transgenic tilapia to *V. vulnificus* infection. Several pathways associated with inflammation, immune response, chemokine and cytochrome were specifically altered in D56-transgenic tilapia, as revealed by KEGG pathway and GO analysis of the wild-type and D56-transgenic tilapia at the baseline (0 hpi) and after infection (6 and 24 hpi). These results suggest that D56 may modulate pro-inflammatory cytokines, AMPs and immune response genes to enhance resistance to *V. vulnificus* (Fig. 8).

## Methods

### Tilapia fish and culture

Tilapia fish *Oreochromis niloticus* were acclimatized in FRP tanks with 2000 l capacity under the controlled conditions of 28 °C with a 13 h light/ 11 h dark cycle with Alanine rich food at 4% body weight per day for 45 days. Wild-type and transgenic tilapia fish with dual expression of delta-6 desaturase in liver and delta-5 desaturase in muscle (D56) were gift from Dr. Jen-Leih Wu’s lab from Institute of Cellular and Organismic Biology, Academia Sinica, Taipei, Taiwan [15]. After 45 days the wild-type tilapias reached an average of 8.93 ± 0.98 cm length and 11.52 ± 3.55 g weight. The transgenic tilapias were 10.06 ± 1.02 cm in length and 16.25 ± 3.61 g weight. The experimental fish were handled after getting approval from the Institutional Animal Care and Use Committee (IACUC) at Academia Sinica, Taiwan, and the IACUC guidelines were followed. According to the formula: n = (Z_1-beta * sqrt(p1q1 + p2q2) + Z_1-alpha/2 * sqrt(2 * p_avg * q_avg))^2 / delta^2, p2 = p1 + delta, p_avg = (p1 + p2) / 2, q_avg = 1 - p_avg. The minimum number of samples in each group was 5 (K = 2, α = 0.05, 1-β = 1–0.2 = 0.8, p1 = 37% and Δ = 74%). A total of 66 animals were used in this study (5 samples * 2 groups * 6 time-points = 60 for qPCR analysis; 6 animals were used for transcriptome analysis). The method for euthanasia was rapid chilling by soaking the animal in ice for 10 min. The ratio of ice to water was roughly 5:1. Ice and water were both measured volumetrically for a total volume of roughly 10 L [41].

### Expression of D56 in transgenic Tilapia

The vector construct and expression of D56 has been explained previously [15]. In brief the delta-6 desaturase transgenic line driven by muscle-specific *CKMb* promoter express the *Fadsd6* with TcFP11 reporter was created by co-injecting the plasmid into the embryo. Similarly delta-5 desaturase transgenic line driven by liver-specific *Fabp10* promoter to express the *Fadsd5* with TcFP13 reporter was created separately. The delta 5 and delta 6 transgenic tilapia have been crossed and the offspring carrying D56 has been selected for the experiment. Atlantic salmon (*Salmon salar*) delta-6 desaturase gene *Fadsd6* and delta-5 desaturase gene *Fadsd5* were used in the construct.

### *V. vulnificus* culture and infection in to tilapia

The pathogen *V. vulnificus* strain 93 U204 was initially cultured in a TCSB (Thiosulfate Citrate Bile Salts Sucrose, BD Difco™) agar plate for 16 h at 28 °C [3]. From overnight cultured plate a single colony was picked and cultured in 3 ml TSB (Tryptone Soy Broth, BD Difco™) with 1.5% NaCl in an incubated shaker at 28 °C with 200 rpm up to four hours until the OD_600mm_ reached 0.7 to 0.9. At the end of acclimatization the wild-type and transgenic fish were subjected to *V. vulnificus* challenge by *i.p.* injecting 50 μl of diluted TSB containing 1.9 × 10^2^ CFU of *V. vulnificus* for each fish. Thirty fishes in each group was infected with *V. vulnificus* and five fish per each group was sacrificed at 0, 3, 6, 12, 24 and 48 h post infection for molecular analysis.

### Extraction of total RNA

The fish was sacrificed at the end of each time point of the experiment and liver tissue were collected for total RNA extraction. The liver tissue was homogenized and total RNA was extracted using Trizol® Reagent by following the company protocol (Invitrogen, USA). Purified RNA was quantified at OD260nm using a ND-1000 spectrophotometer (NanodropTechnology, USA) and qualitated by using a Bioanalyzer 2100 (Agilent Technology, USA) with RNA 6000 LabChip kit (Agilent Technology, USA).

### RNA library construction and transcriptome analysis

RNA extracted from three fish in each group was used for the RNA library construction. Illumina’sTruSeq Stranded Total RNA Library Prep Gold Kit (Cat. 20,020,598) was used for library construction. AMPure XP beads (Beckman Coulter, USA) kit was used for size selection. The sequence was determined using Illumina’s sequencing-by-synthesis (SBS) technology (Illumina, USA) (150 bp, pair-end, 6Gb). Bcl2fastq v2.20 (Illumina, USA) software was used to trim adapter sequence, eliminating Unique Molecular Identifier (UMI) sequences and conversion of BCL files to the per-read FASTQ format. Both adaptor clipping and sequence quality trimming were performed using Trimmomatic v0.36 with a sliding-window approach. After FASTQC, raw reads were aligned to the downloaded tilapia reference genome using HISAT2 software. Differential expression analysis was performed using cuffdiff (cufflinks v2.2.1) with genome bias detection/correction and Welgene Biotech’s in-house pipeline (Supplementary Figure S1). Functional enrichment assay in differentially expressed genes of each experiment design was performed using clusterProfiler v3.6. Genes with low expression level (< 0.3 FPKM value) in either or both of the treated and control samples were excluded (Supplementary File S1). Genes with *p* value ≤0.05 and ≥ 2-fold changes were considered significantly differentially expressed.

### Gene ontology (GO) and Kyoto encyclopedia of genes and genomes (KEGG) analysis

GO enrichment analysis of the differentially expressed genes (DEGs) was implemented using the clusterProfiler package. Pathway enrichment was used for KEGG analysis (https://www.genome.jp/kegg/), which uses public databases to examine *Oreochromis niloticus* biological pathways. The datasets generated and analyzed during the current study are available in the NCBI repository, https://www.ncbi.nlm.nih.gov/bioproject/705417 (Accession: PRJNA705417 ID: 705417) (Accession: PRJNA705417 ID: 705417).

### Gene expression analysis with qPCR

cDNA was obtained from 0.5 μg of total RNA using ReverTra Ace® qPCR RT Master Mix with gDNA Remover kit (TOYOBO, Japan). qPCR was performed using Applied BiosystemsStepOnePlus™ System (ABI, USA) machine. The qPCR reaction mix comprise of 5 μl SYBR® Green Realtime PCR Master Mix (TOYOBO, Japan), 0.5 μl (10 μM) each of gene specific primers (Supplementary Table S1), and 4 μl of twenty times diluted cDNA. Amplification was performed with an initial 95 °C for 1 min and 40 cycles of 95 °C for 15 s, 60 °C for 15 s and 72 °C for 45 s (Supplementary Figure S2). Gene expression relative to *EF-1α* was estimated by 2_T_^-△△C^ method as described [42].

### Statistical analysis

Data are presented as the mean ± standard error of mean (SEM). Statistical analysis was performed using Student’s *t*-TEST (*P < 0.05, **P < 0.01, ***P < 0.001).

## Supplementary Information

**Additional file 1: Supplementary Figure S1.** Analysis of sample correlation.

**Additional file 2: Supplementary Figure S2.** Melting curve analysis of target genes.

**Additional file 3: Supplementary Table S1.** List of primers used in qPCR.

**Additional file 4: Supplementary Table S2.** Comparison of RNA-seq and qRT-PCR.

**Additional file 5: Supplementary File S1.** DEG between D56 and WT.

## Data Availability

The datasets generated and analyzed during the current study are available in the NCBI repository, https://www.ncbi.nlm.nih.gov/bioproject/705417 (Accession: PRJNA705417 ID: 705417).

## Abbreviations

ACKR4: Atypical chemokine receptor 4
ApoA4b: Apolipoprotein A-IV-b
BPI: Bactericidal permeability-increasing protein
CFD: Complement factor D
CFHR1: Complement factor H-related protein 1
CPT1: Carnitine O-Palmitoyltransferase 1
HNF4A: Hepatocyte nuclear factor 4, Alpha
IL-1β: Interleukin 1 beta
LEAP2: Liver-expressed antimicrobial peptide 2
NFκB2: Nuclear factor kappa B subunit 2
NFκBI: NF-kappa-B inhibitor alpha
TH: Tilapia hepcidin
TIMP2: Tissue inhibitor of metalloproteinases 2
TLR-2: Toll- like receptor 2
TLR-5: Toll- like receptor 5
TP: Tilapia piscidin
TNF-α: Tumor necrosis factor alpha
PCK1: Phosphoenolpyruvate carboxykinase 1
PGRN: Progranulin
PPARα: Peroxisome proliferator activated receptor alpha
PRDX1: Peroxiredoxin-1
