# Supplementary material for: Comparative transcriptome analysis reveals ectopic delta-5 and delta-6 desaturases enhance protective gene expression upon Vibrio vulnificus challenge in Tilapia (Oreochromis niloticus)
Source: BMC Genomics. 2021 Mar 22;22:200. doi: 10.1186/s12864-021-07521-5 (PMC7983300; doi:10.1186/s12864-021-07521-5)
Supplement: Supplementary file 1 — Additional file 1: Supplementary Figure S1. Analysis of sample correlation. [file 12864_2021_7521_MOESM1_ESM.doc]

(a) (b)


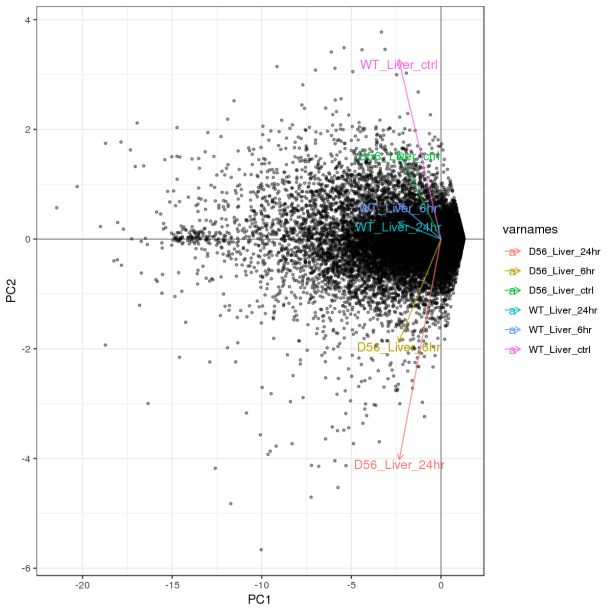

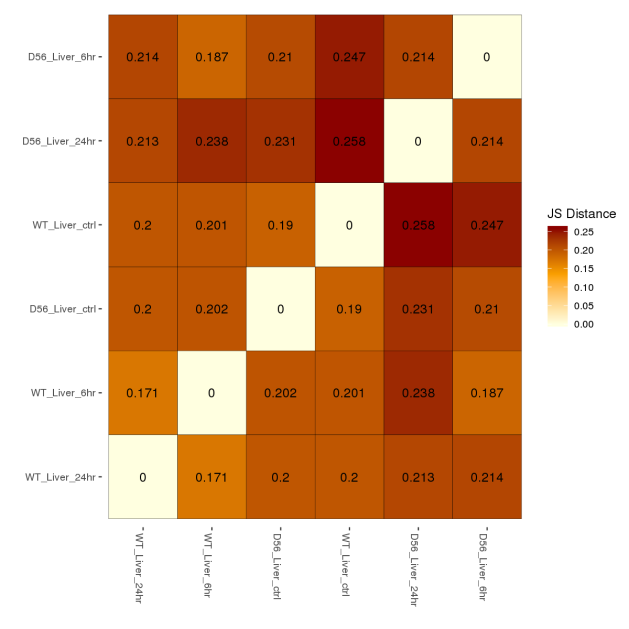


Supplementary Figure S1. Analysis of sample correlation. (a) PCA and (b) JS Distance Map to estimate the correlation between samples.
