# Supplementary material for: Comparative transcriptome analysis reveals ectopic delta-5 and delta-6 desaturases enhance protective gene expression upon Vibrio vulnificus challenge in Tilapia (Oreochromis niloticus)
Source: BMC Genomics. 2021 Mar 22;22:200. doi: 10.1186/s12864-021-07521-5 (PMC7983300; doi:10.1186/s12864-021-07521-5)
Supplement: Supplementary file 2 — Additional file 2: Supplementary Figure S2. Melting curve analysis of target genes. [file 12864_2021_7521_MOESM2_ESM.doc]

a. b. c.


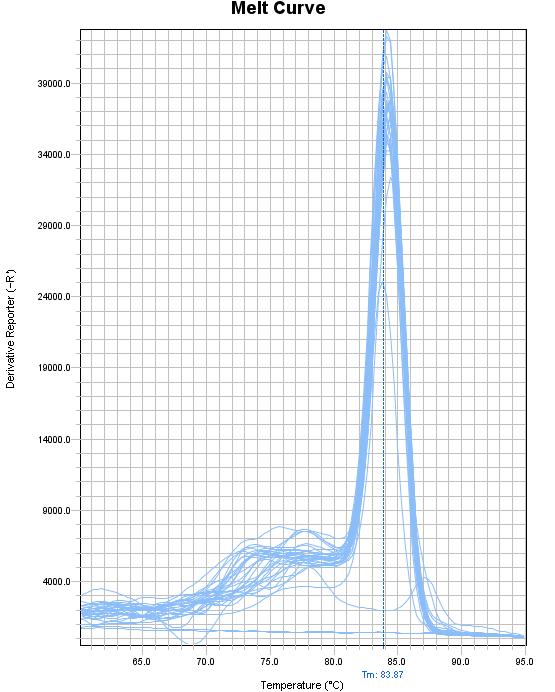

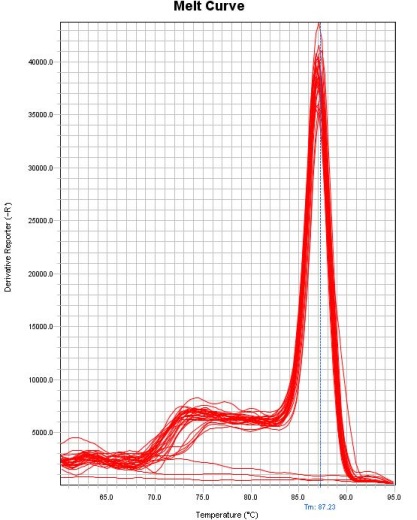

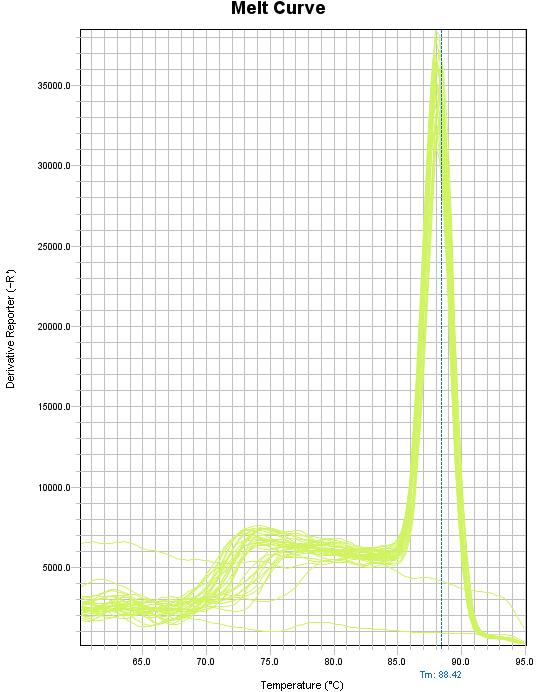


d. e. f.


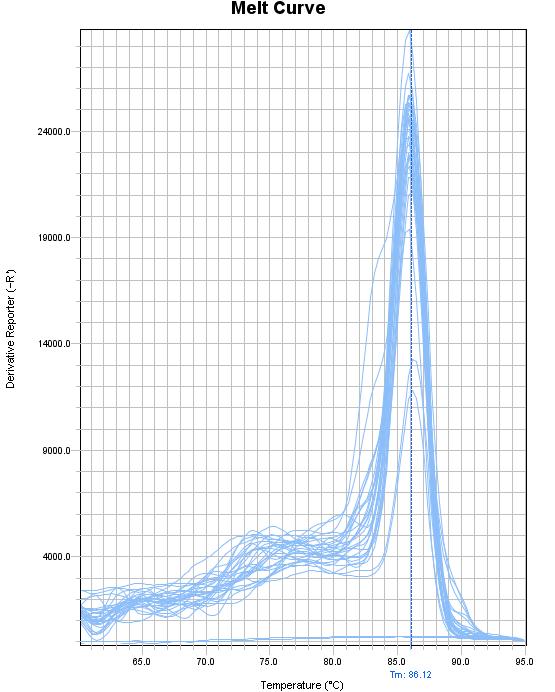

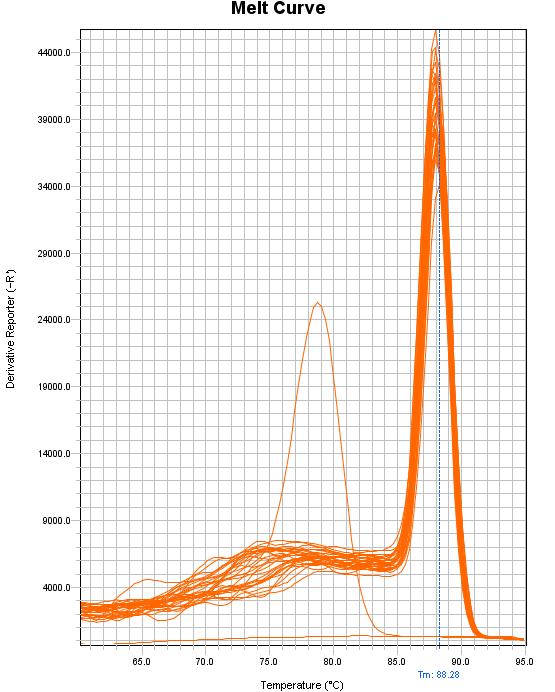

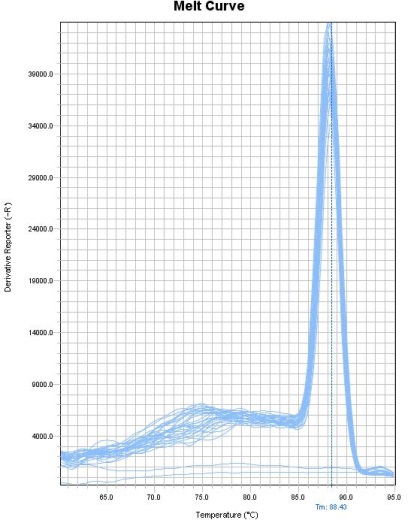


g. h. i.


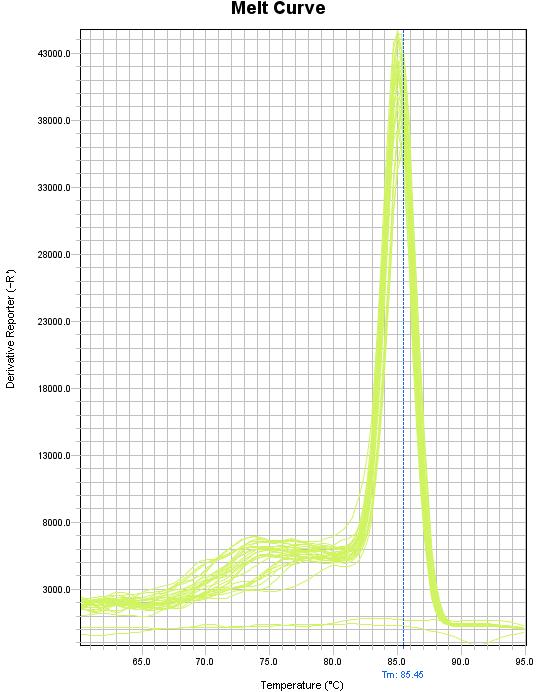

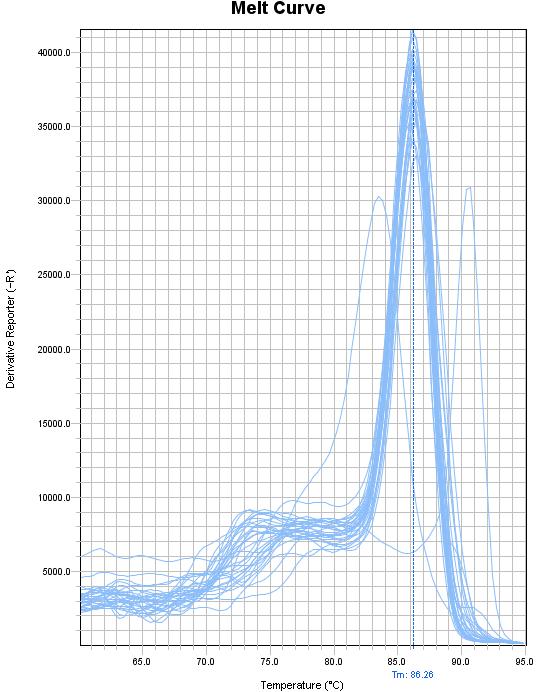

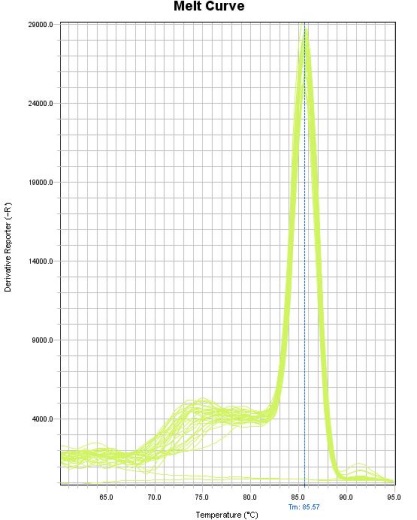


j. k. l.


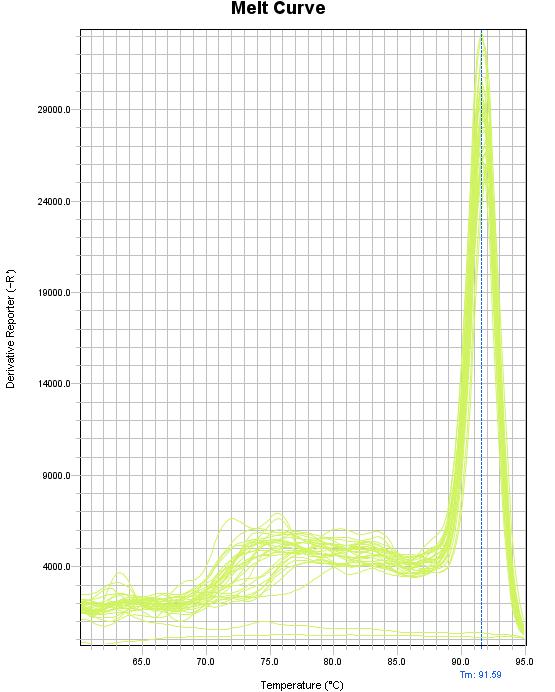

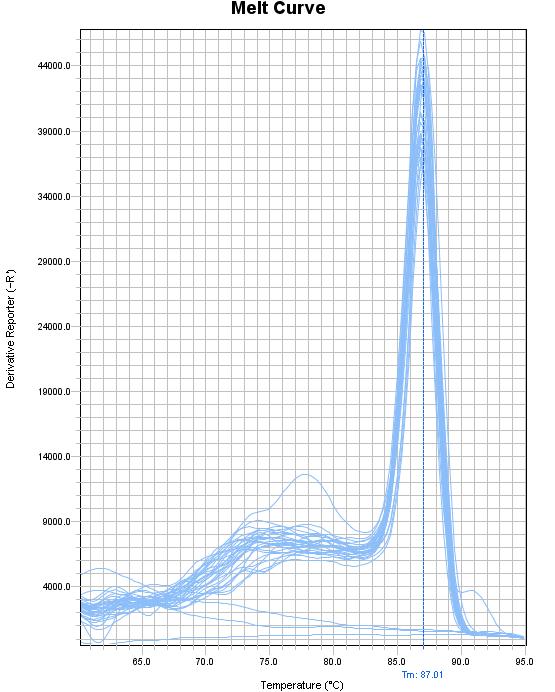

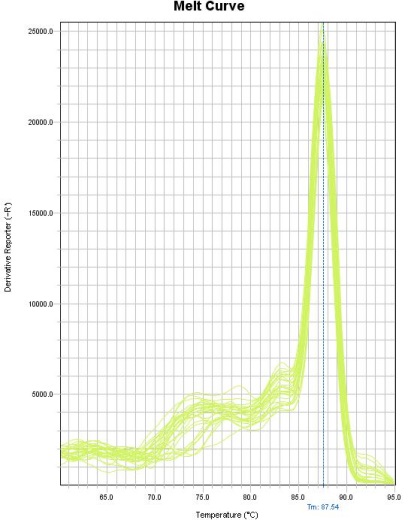


m. n. o.


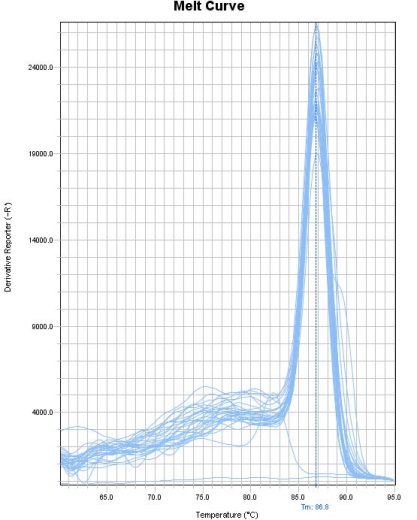

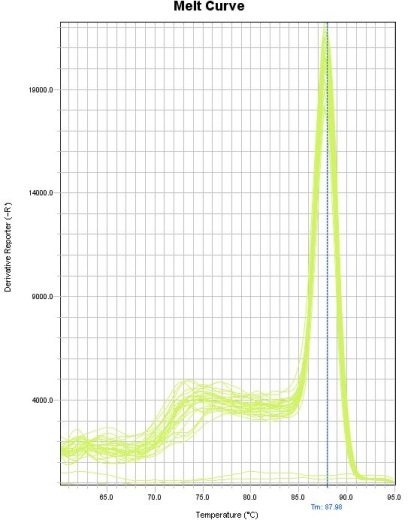

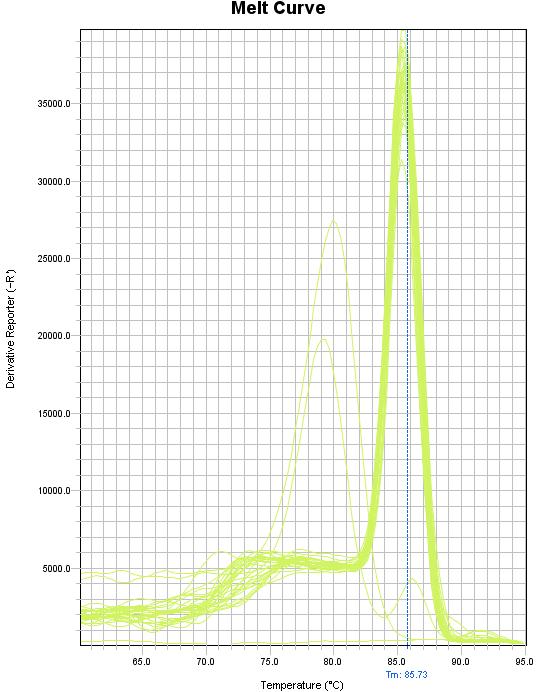


p. q. r.


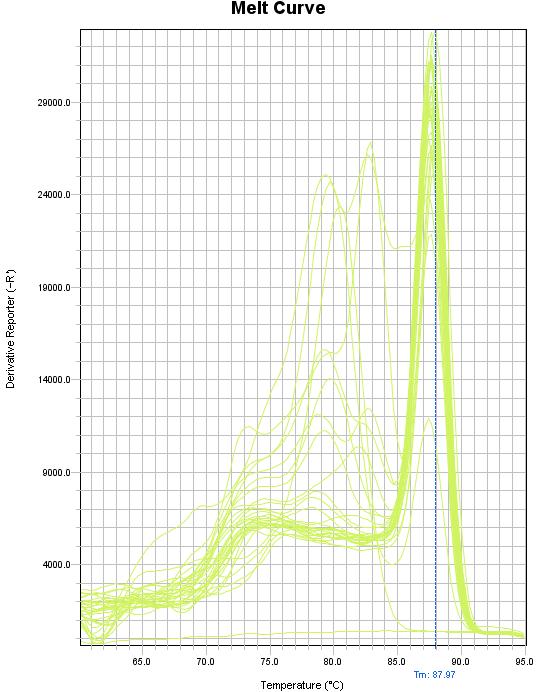

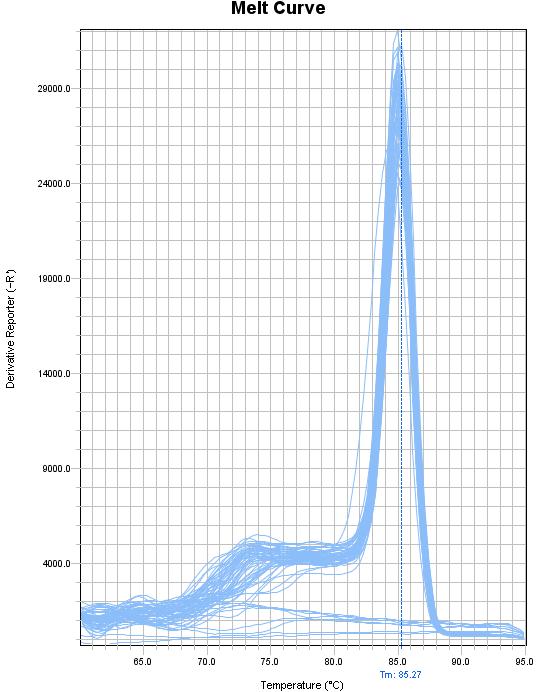

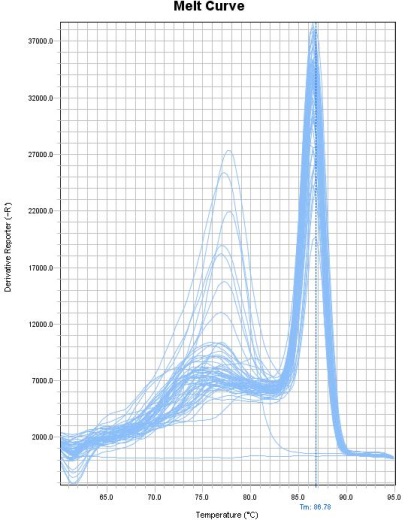


s. t. u.


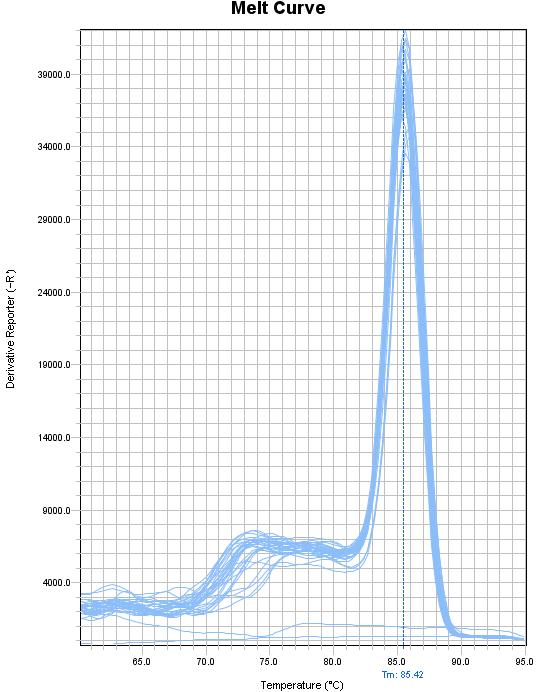

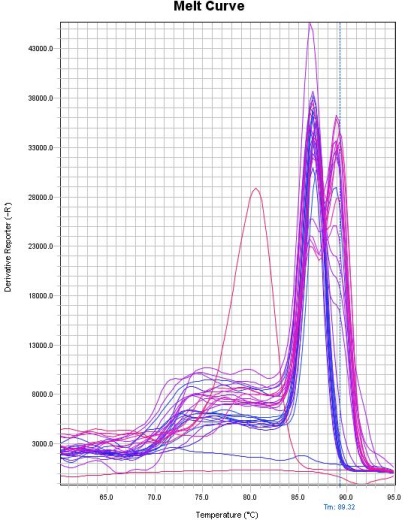

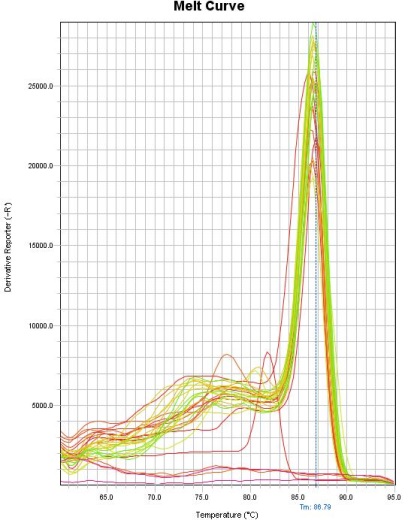


v. w. x.


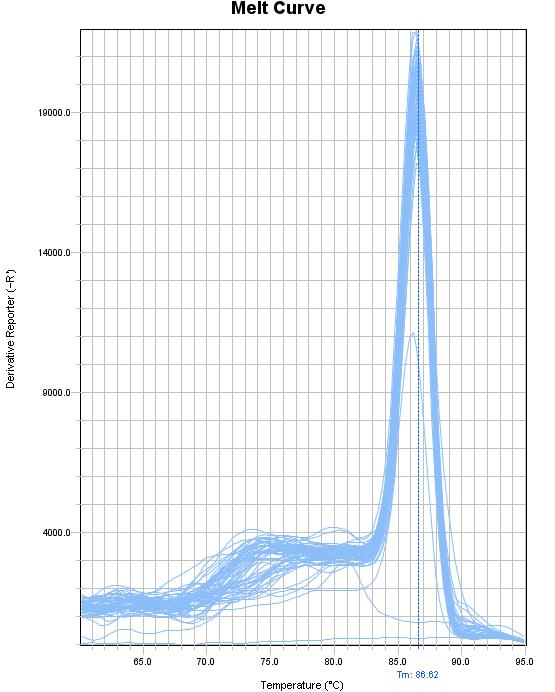

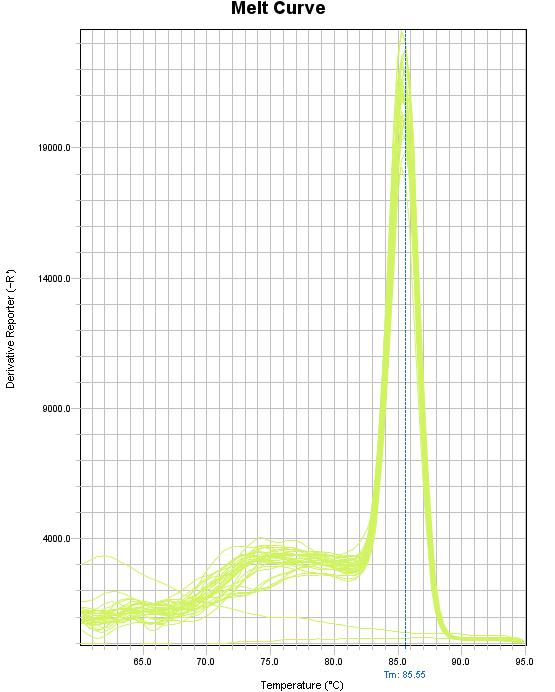

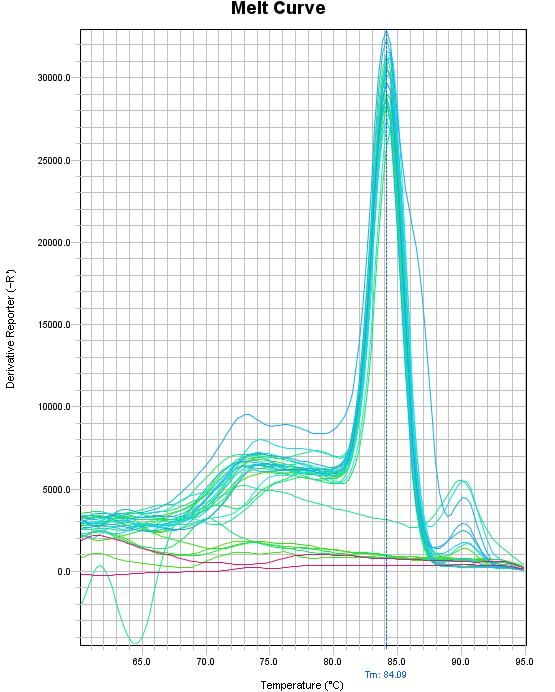


y.


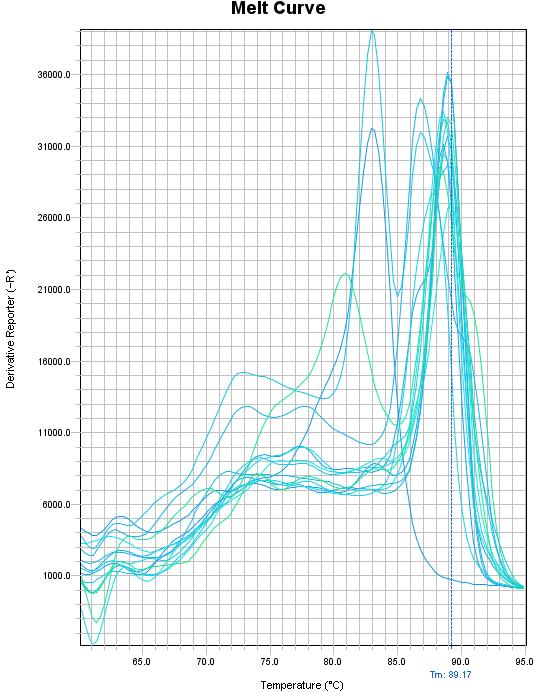


**Supplementary Figure S2.** Supplementary Figure S2. Melting curve analyses of target genes. (a. *CPT1*, b. *PCK1*, c. *ApoA4b*, d. *LEAP2*, e. *EF-1α*, f. *C1qb*, g. *CFHR1*, h. *CFD*, i. *NFκBI*, j. *NFκB2*, k. *TLR-5*, l. *TH*, m. *IL-1β*, n. *ACKR4*, o. *PRDX1*, p. *TIMP2*, q. *BPI*, r. *PGRN*, s. *TP3*, t. *TLR-2*, u. *TNF-α*, v. *HNF4A*, w. *PPARα*, x. *TP4*, y. *TP5*).
