# Supplementary material for: Comparative transcriptome analysis reveals ectopic delta-5 and delta-6 desaturases enhance protective gene expression upon Vibrio vulnificus challenge in Tilapia (Oreochromis niloticus)
Source: BMC Genomics. 2021 Mar 22;22:200. doi: 10.1186/s12864-021-07521-5 (PMC7983300; doi:10.1186/s12864-021-07521-5)
Supplement: Supplementary file 3 — Additional file 3: Supplementary Table S1. List of primers used in qPCR. [file 12864_2021_7521_MOESM3_ESM.doc]

Supplementary Table S1. List of primers used in qPCR.

| Name | 5’→3’ | annotation |
| --- | --- | --- |
| On-CPT1-F | AGCTGTGTTTCTGTGTCCCG | XM_003446465.5 (https://www.ncbi.nlm.nih.gov/nuccore/XM_003446465.5) |
| On-CPT1-R | TCTGCTCTTAACTGGCAAGCA |  |
| On-PCK1-F | TGGAAGGTCGAATGTGTGGG | XM_003448375.4 (https://www.ncbi.nlm.nih.gov/nuccore/XM_003448375.4) |
| On-PCK1-R | GTTGCCATTGCATTGGGGTT |  |
| On-apoa4b-F | GCTTGGTCCCTACACTGACG | XM_003450492.5 (https://www.ncbi.nlm.nih.gov/nuccore/XM_003450492.5) |
| On-apoa4b-R | GTAGGGGGTGGCCATTTCTT |  |
| On-LEAP2-F | GCGTAGCTCACAAACCAAGC | XM_013270789.3 (https://www.ncbi.nlm.nih.gov/nuccore/XM_013270789.3) |
| On-LEAP2-R | TAAGCCATGCCAGTCGTCAG |  |
| On-EF1a-F | TCAACATCGTGGTCATTGG | AB075952 (https://www.ncbi.nlm.nih.gov/nuccore/AB075952) |
| On-EF1a-R | CTCAGCCTTCAGTTTGTCC |  |
| On-C1qb-F | CTTCATCTCCGCTCCCCATC | XM_003448363.5 (https://www.ncbi.nlm.nih.gov/nuccore/XM_003448363.5) |
| On-C1qb-R | CCCATGAGTGCCTGGTATCC |  |
| On-CFHR1-F | GGTTCTACTCTGCTCTGCTTT | XM_005475971.3 (https://www.ncbi.nlm.nih.gov/nuccore/XM_005475971.3) |
| On-CFHR1-R | TTGCCCACCAATCCTCCAC |  |
| On-CFD-F | GTCAAAGGTGCTCGTGGGTG | XM_003447771.5 (https://www.ncbi.nlm.nih.gov/nuccore/XM_003447771.5) |
| On-CFD-R | CTCCCTGCCACCGATTATGC |  |
| On-NFkbI-F | TGAAAGCGAATCGGACGACA | XM_003440691.4 (https://www.ncbi.nlm.nih.gov/nuccore/ XM_003440691.4) |
| On-NFkbI-R | GGAGCCGTGGTTTCGACTAA |  |
| On-NFkb2-F | CACTACAAGCTGGGTGGAGG | XM_003457469.5 (https://www.ncbi.nlm.nih.gov/nuccore/ XM_003457469.5) |
| On-NFkb2-R | GATCGCAGAAGGATGGCACT |  |
| On-TL5-F | CATAGCCTCGCCTAACCCTG | XM_019345473.2 (https://www.ncbi.nlm.nih.gov/nuccore/ XM_019345473.2) |
| On-TL5-R | CCTCACGTTGGTCTCGTTCA |  |
| On-hepcidin-F1 | GACACAAGCGTGGCATCAAG | XM_019365122.2 (https://www.ncbi.nlm.nih.gov/nuccore/ XM_019365122.2) |
| On-hepcidin-R1 | GTTGAGGCAGTAACTGAGGACA |  |
| On-IL1b-F1 | GTTCACCAGCAGGGATGAGATT | XM_019365842.2 (https://www.ncbi.nlm.nih.gov/nuccore/ XM_019365842.2) |
| On-IL1b-R1 | TGCGGTCTTCACTGCCTCC |  |
| On-ACKR4-F | CCTTCATGGGGTCGTCCTTC | XM_003439216.5 (https://www.ncbi.nlm.nih.gov/nuccore/ XM_003439216.5) |
| On-ACKR4-R | TCCGACTGGGATTGGGTTTG |  |
| On-PRDX1-F | GTGATGCCGGATGGACAGTT | XM_003453360.5 (https://www.ncbi.nlm.nih.gov/nuccore/ XM_003453360.5) |
| On-PRDX1-R | GGCGATGACCTCACATCCAA |  |
| On-TIMP2-F | TTTGGCGAGTGCAACAACTG | XM_003450129.5 (https://www.ncbi.nlm.nih.gov/nuccore/ XM_003450129.5) |
| On-TIMP2-R | CCACCTCTTGCTTTCCGACT |  |
| On-BPI-F1 | TCCACTGCCCACAATTGGAA | XM_019358593.2 (https://www.ncbi.nlm.nih.gov/nuccore/ XM_019358593.2) |
| On-BPI-R1 | GCTTTTTCTTGGAAATTTCTGCCA |  |
| On-PGRN-F | TATCCTTTCCTCCGCAAGCC | NM_001279700.1 (https://www.ncbi.nlm.nih.gov/nuccore/NM_001279700.1) |
| On-PGRN-R | TTACCATAACGCAGGAGGGG |  |
| On-TP3-F | GGCAAACATATCCACAGCCTC | <https://doi.org/10.1016/j.fsi.2017.08.041> [30] |
| On-TP3-R | TAAAAGCAGCCCTTTCCCGT |  |
| On-TLR2-F | CAGCCATTGACTCTCTGCCT | <https://doi.org/10.1016/j.fsi.2020.05.017> [30] |
| On-TLR2-R | CACCAGTGGCATGACCTTCA |  |
| On-TNFa-F | GAACACTGGCGACAAAACAGA | https://doi.org/10.1016/j.fsi.2020.05.017 |
| On-TNFa-R | TTGAGTCGCTGCCTTCTAGA |  |
| On-HNF4A-F | TCACTGGGCGGAGCTAGAC | XM_003457051.5 (https://www.ncbi.nlm.nih.gov/nuccore/XM_003457051.5) |
| On-HNF4A-R | CGCTGTAGTCTGCCATGTCC |  |
| On-PPARa-F | CGTATGCCCCAGGCAGAAAA | NM_001290066.1 (https://www.ncbi.nlm.nih.gov/nuccore/ NM_001290066.1) |
| On-PPARa-R | TGAAAGGCGGCTTGCTAGT |  |
| On-TP4-F1 | CTGGGGAAGGCTTTATTCACC | <https://doi.org/10.1016/j.fsi.2017.08.041> [30] |
| On-TP4-R1 | CGTCTACGTCGTATGAGGCG |  |
| On-TP5-F | GGTCCTCTTCATGGCTGATCC | JX006074.1 (https://www.ncbi.nlm.nih.gov/nuccore/JX006074.1) |
| On-TP5-R | CAGGCTTAGCCACACTCTGAA |  |
