# Supplementary material for: Comparative transcriptome analysis reveals ectopic delta-5 and delta-6 desaturases enhance protective gene expression upon Vibrio vulnificus challenge in Tilapia (Oreochromis niloticus)
Source: BMC Genomics. 2021 Mar 22;22:200. doi: 10.1186/s12864-021-07521-5 (PMC7983300; doi:10.1186/s12864-021-07521-5)
Supplement: Supplementary file 4 — Additional file 4: Supplementary Table S2. Comparison of RNA-seq and qRT-PCR. [file 12864_2021_7521_MOESM4_ESM.doc]

|  | Transcriptome-0h/6h/24h(Log2Ratio) | Real-time PCR-0h/6h/24h(Log2Ratio) |
| --- | --- | --- |
| *On-CPT1* | 4.18/4.13/4.22 | 0.42/0.6/1.73 |
| *On-PCK1* | 3.74/1.05/3.42 | -0.54/2.08/2.36 |
| *On-ApoA4b* | 5.4/1.79/0.74 | 0.05/0.37/-1.26 |
| *On-LEAP2* | 1.05/2.22/3.88 | -0.87/0.07/1.16 |
| *On-C1qb* | 1.61/-0.47/0.74 | -0.04/1.19/-1.26 |
| *On-CFHR1* | 0.99/2.06/2.15 | 0.76/1.99/1.93 |
| *On-CFD* | -0.43/-0.04/-1.75 | 0.54/1.19/-0.15 |
| *On-NFκBI* | 0.2/1.14/1.94 | 0.28/0.35/2.2 |
| *On-NFκB2* | 0.12/0.99/2.42 | -0.31/0.81/1.3 |
| *On-TLR-5* | 0.81/1.58/3.72 | 0.96/1.71/7.82 |
| *On-TH* | 0/2.76/1.24 | 0.55/0.94/6.51 |
| *On-IL-1β* | 2.75/-0.45/6.16 | 1.05/0.24/5.1 |
| *On-ACKR4* | 1.51/3.4/2.82 | -0.15/0.51/0.33 |
| *On-PRDX1* | 1.16/2.93/2.71 | 2.38/2.33/0.59 |
| *On-TIMP2* | 0.48/3.7/4.77 | 0.21/1.4/6.38 |
| *On-BPI* | 2.87/0.07/6.07 | 1.08/1.18/2.54 |
| *On-PGRN* | 5.77/0.44/4.47 | 2.29/5.28/0.03 |
| *On-TP3* | - | -0.27/1.24/-2.05 |
| *On-TLR-2* | - | 1.64/2.9/-2.39 |
| *On-TNF-α* | 0.82/0.49/3.17 | 3.12/-0.96/-0.05 |
| *On-HNF4A* | -0.19/-2.2/-0.61 | 0.75/1.06/-0.7 |
| *On-PPARα* | 0.47/-1.17/-1.09 | 0.09/0.94/-1.9 |
| *On-TP4* | - | -1.27/-4.68/-6.19 |
| *On-TP5* | - | -3.74/1.84/-0.84 |

Supplementary Table S2. Supplementary Table S2. Comparison of RNA-seq and qRT-PCR.
